# Ambivalence and Interpersonal Liking: The Expression of Ambivalence as Social Validation of Attitudinal Conflict

**DOI:** 10.3389/fpsyg.2020.525301

**Published:** 2020-09-29

**Authors:** Daniel Toribio-Flórez, Frenk van Harreveld, Iris K. Schneider

**Affiliations:** ^1^Research Group ‘Moral Courage’, Max Planck Institute for Research on Collective Goods, Bonn, Germany; ^2^School of Education, Technical University of Munich, Munich, Germany; ^3^Department of Psychology, University of Amsterdam, Amsterdam, Netherlands; ^4^Department of Psychology, University of Cologne, Cologne, Germany; ^5^Center for Social and Economic Behavior, University of Cologne, Cologne, Germany

**Keywords:** ambivalence, interpersonal liking, attitude similarity, social validation, attitudinal conflict, self-presentation

## Abstract

Literature on attitude similarity suggests that sharing similar attitudes enhances interpersonal liking, but it remains unanswered whether this effect also holds for ambivalent attitudes. In the present research, we shed light on the role attitudinal ambivalence plays in interpersonal liking. Specifically, we examine whether people express ambivalence strategically to generate a positive or negative social image, and whether this is dependent on the attitudinal ambivalence of their perceiver. We test two alternative hypotheses. In line with the attitude-similarity effect, people should express ambivalence toward ambivalent others to enhance interpersonal liking, as sharing ambivalence might socially validate the latter’s experience of attitudinal conflict. On the other hand, people might express more univalence, as ambivalence may drive ambivalent others toward the resolution of their attitudinal conflict, and univalent stances could help to achieve that goal. In two studies (*N* = 449, 149), people expressed similar attitudes to those of their perceivers, even when the latter experienced attitudinal conflict (Studies 1 and 2). Moreover, they composed an essay, the message of which validated their perceiver’s attitudinal conflict (Study 2). In line with these results, we further observe that the more people experienced their ambivalence as conflicting, the more they liked others who similarly experienced attitudinal conflict (Study 1). These findings suggest that the expression of ambivalence can have important interpersonal functions, as it might lead to an enhanced social image when interacting with those coping with attitudinal conflict.

## Introduction

Imagine two individuals, Sam and Robin, who meet for the first time and engage in a conversation about nuclear disarmament. During the conversation, Sam learns that Robin holds a favorable view toward nuclear disarmament. If Sam aimed to make a good impression on Robin, his best bet would probably be to express a similarly positive opinion about the topic. In contrast, Sam could presumably share an opposing negative view if his goal was to be negatively perceived by Robin. These rather intuitive predictions are supported by extensive research on the so-called “attitude-similarity” effect (e.g., Singh et al., 2008; Reid et al., 2013; Sprecher, 2014).

Most of the work on attitude similarity focuses on situations where individuals hold one-sided or *univalent* attitudes (e.g., if Sam and Robin support *or* oppose nuclear disarmament). However, reality is often more complex, and most people hold two-sided or *ambivalent* attitudes toward a wide variety of attitude objects, e.g., immigration (Reyna et al., 2013), gender roles (Sjöberg, 2010), gay rights (Garner, 2013), geopolitics (Stoeckel, 2013), green products (Chang, 2011), or meat consumption (Onwezen and van der Weele, 2016). Despite the ubiquitous nature of ambivalent attitudes, the role that ambivalent attitudes play in interpersonal liking remains widely unexplored.

The present work aims to shed light on what Sam would do in order to make a favorable impression on Robin, if the latter held an ambivalent attitude about nuclear disarmament. Put differently, we investigate whether people are able to infer the similarity–dissimilarity preferences of ambivalent others and adjust their attitude expression to enhance or depreciate their own interpersonal liking. Thus, we intend to contribute both to the literature on ambivalence as well as to the literature on attitude similarity by examining the strategic expression of ambivalent attitudes in the context of interpersonal liking and self-presentation.

Research on attitude similarity consistently shows that sharing similar attitudes toward different topics (e.g., disliking modern art, opposing socialism, and enjoying science fiction) predicts interpersonal liking (Byrne, 1961; Byrne and Nelson, 1965b; Byrne et al., 1968a), even over and above other factors related to interpersonal attraction, such as physical attractiveness (Byrne et al., 1968b). With regard to its generalizability, the attitude-similarity effect has been further observed in children (Fawcett and Markson, 2010) and adolescents (Cavior and Dokecki, 1973) and also across different cultures (Byrne et al., 1971). Its most influential explanatory framework is the *reinforcement theory of attraction* (Byrne, 1961; Byrne and Nelson, 1965a). This theory postulates that those who share similar attitudes are likely to be perceived as more attractive, because they induce positive affect through the reinforcement or validation of one’s own beliefs, opinions, or feelings. Recent research supports this notion by showing that the relationship between attitude similarity and interpersonal liking is indeed sequentially mediated by feelings of validation and positive affect (Singh et al., 2017).

The attitude-similarity effect has implications for self-presentation contexts (e.g., a first date, a job interview, or a political debate), where people aim to make a good impression on others. In these situations, people may strive to convey attitude similarity in order to enhance their social image, leading them strategically to express attitudes similar to those of their perceivers. Previous work has emphasized the social value of attitude similarity in self-presentation settings. In one study, people presented themselves to a third party based on their evaluation of a partner who held similar (vs. dissimilar) attitudes. When asked to make a good impression on the third party, people valued the partner who shared similar (vs. dissimilar) attitudes more positively (Jellison and Oliver, 1983). This finding suggests that people are conscious about the social value that attitude similarity has for others, and that this affects their strategy when aiming to project a positive social image.

Attitude-similarity literature has mainly focused on univalent attitudes while overlooking ambivalent attitudes. This is not surprising given that researchers became interested in ambivalence only relatively recently (Thompson et al., 1995; Jonas et al., 1997). In psychology, ambivalence generally refers to simultaneous positive and negative evaluations of the same attitude object (Jonas et al., 2000). Research on ambivalence distinguishes two conceptualizations of this construct (cf. van Harreveld et al., 2015), and their different motivational and behavioral implications give rise to different predictions with respect to interpersonal liking.

The first, *Objective Ambivalence* (OA), refers to the structure of the attitude, characterized by the concurrence of positive and negative evaluative components (Kaplan, 1972; Eagly and Chaiken, 1998). Measures of OA take into account both the similarity and strength of the positive and the negative evaluations (Thompson et al., 1995). In line with the attitude-similarity effect, a first possibility is that ambivalent individuals show stronger preferences toward others with a similarly ambivalent attitude structure. The few studies that have investigated ambivalence in the context of interpersonal liking have provided indirect evidence for this notion. For example, a study showed that when presenting two fictitious characters as having a similar ambivalent attitude toward each other (i.e., [Robin] likes and dislikes [Sam] and vice versa), people perceived their relationship as more stable than when their attitudes differed (Miller and Geller, 1972). In a different study, people were asked to report their preference between two targets who expressed either ambivalence (i.e., expressing oneself in part very positively and in part very negatively) or indifference (i.e., expressing oneself neutrally) about several ambivalent attitude objects (e.g., euthanasia). Researchers found that ambivalent participants tended to prefer the ambivalent target relative to the indifferent target (Ullrich and Krueger, 2010). Altogether, these findings suggest that similar levels of OA (i.e., shared attitudinal structure) might cue interpersonal liking.

The second conceptualization, *Subjective Ambivalence* (SA), refers to the meta-emotional response to the perceived inconsistency in the structure of an ambivalent attitude (Priester and Petty, 1996). SA relates to the negative affect often experienced when one recognizes ambivalence as an attitudinal conflict (Newby-Clark et al., 2002; Nordgren et al., 2006). In these cases, people feel motivated to reduce the psychological discomfort (van Harreveld et al., 2009) or to resolve their attitudinal conflict (Clark et al., 2008; Sawicki et al., 2013; DeMarree et al., 2015). These motivational effects of ambivalence have implications in the context of interpersonal liking, as they might determine the interpersonal preferences of those coping with the unpleasantness of ambivalence. In particular, subjectively ambivalent individuals might arguably like others who facilitate the accomplishment of one of these motivated goals, leading to two possible alternatives. On the one hand, subjectively ambivalent individuals might prefer ambivalent others who feel similarly conflicted about the same topic, as the latter could socially validate the affective experience of attitudinal conflict. On the other hand, subjectively ambivalent individuals might like others who facilitate the resolution of their attitudinal conflict, even when these do not necessarily hold a similarly ambivalent attitude. In this respect, univalent others could plausibly become an attractive option as providers of one-sided information, which might help to resolve the ambivalence, and with it, reduce its psychological discomfort.

The proposed alternative (and opposing) predictions about the social preferences of ambivalent individuals also relate to how individuals present themselves when interacting with an ambivalent other. As discussed earlier, the strategic expression of attitudes, including ambivalent attitudes, can be a crucial resource in the enhancement (or depreciation) of one’s social image in a self-presentation context. In the case of ambivalence, previous work shows that people regulate the expression of ambivalent attitudes based on the impression they aim to create on others. Specifically, people reported more ambivalence when they aimed to generate a positive impression, which they seemed to do by default in a baseline condition, in comparison with when they intended to generate a negative impression (Pillaud et al., 2013). The authors argue that the expression of ambivalence can function as a cue of social competence when discussing controversial topics, i.e., when others may appreciate pondered and balanced information about both sides of a controversy (Pillaud et al., 2013, 2018).

We follow up on these results and argue that the expression of ambivalence—and, by extension, of other kinds of attitudes—likely depends on the ambivalence expressed by the perceiver. According to the attitude-similarity literature, the extent to which the expression of attitudes can be an efficient resource for the enhancement (vs. depreciation) of interpersonal liking substantially hinges on the degree of similarity with the perceiver. However, in the case of an ambivalent perceiver and, more concretely, of those who experience ambivalence as an attitudinal conflict, it is not clear whether the expression of similar ambivalence would lead to higher interpersonal liking in contrast to the expression of univalent attitudes. As previously argued, both alternatives could fulfill—or truncate—distinct psychological needs of the perceiver (i.e., validate vs. resolve the attitudinal conflict). If perceivers aim to reduce their experienced discomfort, they should prefer someone who validates their attitudinal conflict, rather than someone who offers one-sided information to resolve it. Alternatively, if perceivers aimed to resolve the attitudinal conflict, they should prefer someone who offered one-sided information to someone who validated the conflict. The present work seeks to provide answers to this matter.

## Research Overview

In two studies, we investigated the expression of ambivalence as a function of the perceiver’s attitudinal profile in a self-presentation setting. We tested two main alternative hypotheses:

**H1.** People express more ambivalence when aiming to be judged positively (vs. negatively) by an ambivalent perceiver.

**H1′.** People express more univalent attitudes when aiming to be judged positively (vs. negatively) by an ambivalent perceiver.

H1 was based on the assumption that the expression of ambivalence would be used as a social validation of the perceiver’s ambivalence, whereas H1′ entailed the intention of resolving the perceiver’s attitudinal conflict through the provision of one-sided information.

Moreover, for perceivers holding univalent attitudes, we expected to replicate patterns of attitude similarity.

Beyond the strategic expression of ambivalence, Study 1 further explored whether ambivalent individuals liked ambivalent (vs. univalent) others. This would clarify whether people’s self-presentation strategy aligned to the similarity–dissimilarity preferences of ambivalent individuals. For its part, Study 2, which was pre-registered, additionally introduced the possibility of analyzing whether the strategic expression of ambivalence was driven by the underlying intention of socially validating the attitudinal conflict of others.

## Study 1

In Study 1, we examined whether people strategically based their attitude expression on their perceiver’s attitude profile, with the goal of enhancing their interpersonal liking. For this purpose, we adapted the “self-presentation paradigm” used by Pillaud et al. (2013). In this paradigm, people express their attitudes about a specific attitude object with the objective of making a positive (i.e., *self-enhancement* condition) or negative (i.e., *self-depreciation* condition) impression on a perceiver. These expressed attitudes are compared with a baseline measure of participants’ attitudes (i.e., *control* condition). In our case, we used the topic of “unrestricted freedom of speech” as attitude object. We chose freedom of speech because it has been argued as eliciting ambivalence (Downs and Cowan, 2012), and because it was of contemporary relevance on the dates of data collection (i.e., June 2017) due to the rise of populist political speeches in Western politics.

In contrast to the original paradigm, where people lacked information about their perceiver, we manipulated the perceiver’s attitudinal profile. We configured three attitudinal profiles by independently manipulating two attitudinal elements: first, the level of OA for comparing between univalent (i.e., low OA) and ambivalent perceivers (i.e., high OA) and second, the level of SA for comparing between ambivalent perceivers with low and high experienced attitudinal conflict. Thus, one of the three different perceivers represented a univalent attitude holder (i.e., low OA) with a *univalent positive* attitude.^1^ This univalent perceiver would allow to reproduce the attitude-similarity effect by observing the expression of similar (vs. dissimilar) attitude valence in the self-enhancement (vs. self-depreciation) condition. The other two perceivers represented ambivalent attitude holders (i.e., high OA) who differed in their level of experienced conflict: low SA or *objectively ambivalent* and high SA or *subjectively ambivalent*. In this case, the distinction between objectively and subjectively ambivalent perceiver allowed to examine whether the aforementioned motivational effects of subjective ambivalence (i.e., inclination toward the reduction of its psychological discomfort vs. the resolution of the attitudinal conflict) implied a qualitative difference that people considered in their self-presentation strategy.

Thus, the study followed a 3 × 3 mixed factorial design, with the self-presentation instructions as within-subject factor (control, self-enhancement, and self-depreciation) and the perceiver’s attitudinal profile as between-subject factor (univalent positive, objectively ambivalent, and subjectively ambivalent). The dependent variables were levels of OA and SA and the attitude valence expressed by participants. Compared with their baseline attitudes, we expected that people would express more OA and less SA toward the objectively ambivalent perceiver in the self-enhancement condition, whereas in the self-depreciation condition, they would express less OA and SA. With regard to the subjectively ambivalent perceiver, we predicted that people would express more OA and SA in the self-enhancement condition, whereas in the self-depreciation condition, the expressed OA and SA will be lower. Lastly, those presenting themselves to the univalent positive perceiver would express more positive (i.e., similar) attitudes in the self-enhancement condition and more negative (i.e., dissimilar) attitudes in the self-depreciation condition.

As a secondary goal, Study 1 aimed to explore whether people’s ambivalence predicted the interpersonal liking of the ambivalent (vs. univalent) perceivers. Therefore, in addition to the self-presentation paradigm, people assessed each perceiver’s interpersonal liking.

### Method

#### Ethics Statement

This study was carried out in accordance with the recommendations and protocol approval of the Ethics Review Board of the Faculty of Social and Behavioral Sciences (FMG) of the University of Amsterdam. All subjects gave written informed consent in accordance with the Declaration of Helsinki.

#### Participants

According to an a priori power analysis using G^∗^Power 3.1.9.2, we estimated a minimum sample size of 264. Significance level, correlation among repeated measures, and non-sphericity correction were kept constant at α = 0.05, *r* = 0.50, and ε = 0.75, respectively. For the expected two-way interaction between the between- and the within-subject factor with a medium effect size (i.e., $\eta_{\text{p}}^{2}$ = 0.09), the estimated sample size for a statistical power of 0.96 was 66 participants. However, to detect a medium effect size in the follow-up between-subject comparisons, we estimated a minimum of 88 participants per group for a 95% statistical power. We recruited an online sample of 581 participants from the online market Crowdflower (currently, Figure Eight), who were paid 0.50 USD (*n* = 544), and from the University of Amsterdam, in this case compensated with 0.25 course credits (*n* = 37). We excluded data from 121 participants who did not answer or missed an attention check for screening out random clicking (i.e., “In this question, we want you to click on number six”), eight who did not complete all the dependent measures, two participants who reported to be underage (i.e., below 18), and one participant from the University of Amsterdam who had participated in a related study. The final sample consisted of 449 participants (57.2% females, *M*_age_ = 35.76, *SD*_age_ = 13.37, ranging from 18 to 76), 84% were from the United States, 7% from the Netherlands, and the remaining 8% from other countries.

#### Procedure

Participants provided informed consent through a Qualtrics survey (Qualtrics, 2018; Provo, UT, United States), which described the study and its focus on personal opinions and feelings about unrestricted freedom of speech. After reporting demographics and creating an anonymous ID code, participants completed a questionnaire including the baseline measures of OA and SA toward unrestricted freedom of speech (i.e., *control* condition).

Next, participants learned that the responses of a randomly selected participant from a previous experiment would be presented to them. The survey simulated a data-transfer process and displayed a random ID, ostensibly identifying a real participant. The responses of this participant appeared on screen, reflecting one of the three manipulated attitudinal profiles (i.e., univalent positive, objectively ambivalent, subjectively ambivalent; see Supplementary Material). Then, participants responded to several manipulation checks assessing whether participants perceived the manipulated attitudinal profiles as intended (e.g., “To what extent do you think the opinion that participant 67A12 has about [attitude object] is *positive* or *negative*/participant 67A12 has a *clear* or *confusing* opinion about [attitude object]/participant 67A12 feels *confident* or *unconfident* about his/her opinion about [attitude object]?”).

After responding to the manipulation checks, the self-presentation manipulation followed. Participants had to respond to the same questionnaire about unrestricted freedom of speech (i.e., measures of OA and SA) with the objective of making a specific impression on participant 67A12. The self-presentation instructions were the same as used by Pillaud et al. (2013). In the *self-enhancement* condition, the instructions read: “As you fill in the questionnaire, we would like you to try to generate a GOOD image of yourself. That is, to answer in such a way as to be judged in a POSITIVE way by participant 67A12.” In the *self-depreciation* condition, the words GOOD and POSITIVE were exchanged for BAD and NEGATIVE, respectively. The order of presentation of both within-subject conditions was counterbalanced.

The last part of this survey consisted of the evaluation of the perceiver’s interpersonal liking. Further exploratory measures of interest in discussing the topic of unrestricted freedom of speech with the perceiver, the perceiver’s relative knowledge and instrumentality, and emotional closeness to the perceiver were part of this last questionnaire and can be accessed in the Supplementary Material. After responding to these questions, participants were debriefed and provided with information for receiving their compensation.

#### Measures

##### Objective ambivalence

We evaluated participants’ OA through a bivariate scale consisting of two 4-point items that asked, respectively, to consider the positive or negative aspects of unrestricted freedom of speech and report how positive or negative the participant evaluated it (1—*not at all positive* or *negative*, 4—*extremely positive* or *negative*). We used the following formula, OA = 1/2 (Positive + Negative) – |Positive – Negative| (Thompson et al., 1995), to calculate an OA index, with higher scores indicating higher OA.

##### Subjective ambivalence

We assessed participants’ SA through the *Subjective Ambivalence Scale* (Priester and Petty, 1996), which consists of three 11-point scales that measured the experienced conflict, indecision, and mixed reactions toward unrestricted freedom of speech (0—*I feel no conflict at all, I feel no indecision at all, Completely one-sided reactions*, 10—*I feel maximum conflict, I feel maximum indecision, Completely mixed-sided reactions*). We computed an SA index by averaging the score of these three items (α = 0.827).

##### Attitudinal valence

We used the bivariate scale from the OA measure to estimate participants’ attitudinal valence and computed the difference between how positive and how negative participants evaluated unrestricted freedom of speech, with higher scores reflecting more positive (vs. negative) attitudes.

##### Interpersonal liking

The perceiver’s interpersonal liking was measured with a single item, “How much would you like this person?”. The item used a 7-point scale (1—*I would not like this person at all*, 7—*I would really like this person*).

### Results

#### Manipulation Checks

Three independent GLM analyses showed that participants distinguished the different perceivers in line with the manipulated attitudinal profiles. Specifically, we observed significant between-subject differences in the extent to which people evaluated their perceiver’s opinion as positive/negative, *F*(2,446) = 17.933, *p* < 0.001, $\eta_{\text{p}}^{2}$ = 0.074, clear/confused, *F*(2,446) = 57.500, *p* < 0.001, $\eta_{\text{p}}^{2}$ = 0.205, and confident/unconfident, *F*(2,446) = 53.779, *p* < 0.001, $\eta_{\text{p}}^{2}$ = 0.194. With the exception of the difference in positivity/negativity between the univalent positive and the objectively ambivalent perceivers (*p* = 0.41), every pairwise comparison reached significance (*p*s ≤ 0.045) after applying Bonferroni corrections. As Table 1 summarizes, the univalent positive and the objectively ambivalent perceivers were perceived significantly more positive in their attitude than the subjectively ambivalent, whereas the latter was perceived as the perceiver with the most confused and the least confident stance, followed by the objectively ambivalent, and ending with the univalent positive.

**TABLE 1:** Estimated mean levels of manipulations checks used to describe the different perceiver’s attitudinal profiles – Study 1.

**Perceiver attitudinal profile**	**Positivity**	**Clearness**	**Confidence**
Univalent positive	5.25 [5.01, 5.48]	5.17 [4.91, 5.44]	5.62 [5.37, 5.88]
Objectively ambivalent	5.00 [4.77, 5.23]	4.71 [4.45, 4.97]	5.01 [4.75, 5.26]
Subjectively ambivalent	4.27 [4.03, 4.51]	3.19 [2.92, 3.46]	3.72 [3.46, 3.98]

*In brackets, lower and upper bounds of 95% CIs. Scales were from 1 to 7.*

#### Ambivalence and Self-Presentation

Our main goal was to disentangle whether the expression of OA, SA, and attitude valence differed across self-presentation conditions as a function of the perceiver’s attitudinal profile. Thus, our main interest was the interaction between self-presentation and attitudinal profile. We tested three independent mixed models that included the fixed effects of the self-presentation instructions and the perceiver’s attitudinal profile, together with their two-way interaction. The models additionally accounted for the within-subject nature of our data by including the participants’ ID as random factor. When any mixed model showed a singular fit, which generally suggests that the model’s random structure overfits the data (Bates et al., 2018), we instead conducted a repeated measures GLM analysis without the random factor. In what follows, we will describe the results for each of the different dependent measures.

##### Objective ambivalence

**FIGURE 1:**
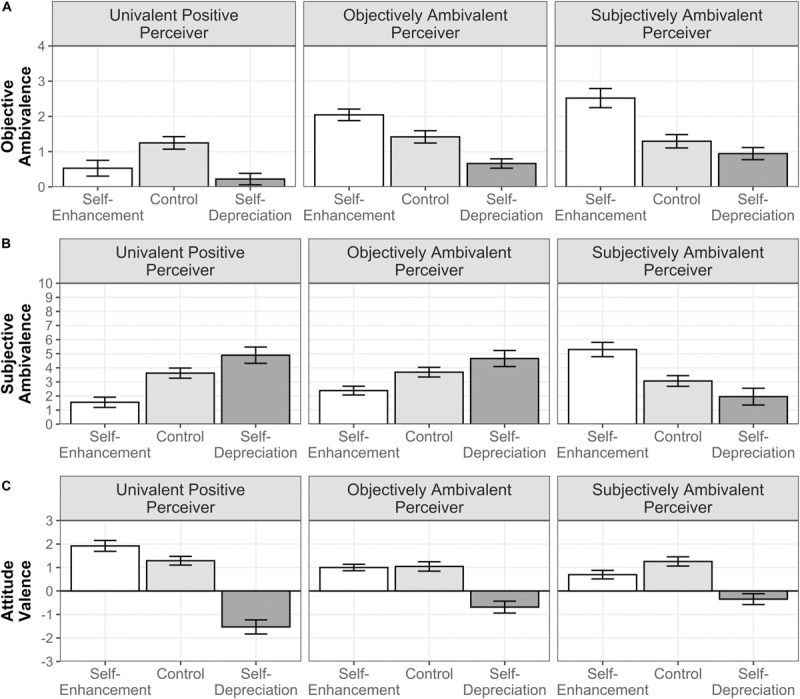
Moderated influence of self-presentation instructions by the perceiver’s attitudinal profile on reported OA **(A)**, SA **(B)**, and attitude valence **(C)** in Study 1. Error bars represent 95% CIs.

The fixed effects of both the self-presentation instructions, *F*(2,892) = 129.090, *p* < 0.001, $\eta_{\text{p}}^{2}$ = 0.185, and the perceiver’s attitudinal profile, *F*(2,446) = 62.286, *p* < 0.001, $\eta_{\text{p}}^{2}$ = 0.086, were significant. Furthermore, the two-way interaction between these two factors was significant, *F*(4,892) = 36.417, *p* < 0.001, $\eta_{\text{p}}^{2}$ = 0.114 (see Figure 1A). We further examined this interaction with simple effect analyses—from here on, every simple effect analysis reported in the manuscript included Bonferroni adjustments for multiple comparisons. In the self-enhancement condition, people who were matched with the objectively and the subjectively ambivalent perceivers expressed significantly higher OA than the baseline (*p*s < 0.001), with slightly higher expressed OA toward the subjectively ambivalent (*p* = 0.015). In contrast, those participants who were matched with the univalent positive perceiver reported a lower level of OA relative to the baseline (*p* < 0.001). In the self-depreciation condition, people significantly reduced their expressed OA toward the objectively ambivalent (*p* < 0.001) and the univalent positive (*p* < 0.001) perceivers in comparison with the baseline, but this was not the case for those matched with the subjectively ambivalent perceiver (*p* = 0.14).

The observed interaction showed a general tendency to express ambivalent (univalent) attitudes similar to the ones expressed by the ambivalent (univalent) perceivers when the aim was to make a good impression, but people generally expressed more dissimilarly univalent attitudes when instructed to make a bad impression. This expression of univalent attitudes in the self-depreciation condition was, in the case of the ambivalent perceivers, a clear dissimilarity from their two-sided attitudes; for the univalent positive perceiver, however, the dissimilarity potentially corresponded to the expression of univalent attitudes of opposite (negative) valence. Our measure of attitude valence would clarify this issue.

##### Subjective ambivalence

The main effect of self-presentation on reported SA was significant, *F*(1.534,683.967) = 24.162, *p* < 0.001, $\eta_{\text{p}}^{2}$ = 0.051, but the effect of the perceiver’s attitudinal profile was not, *F*(2,446) = 0.898, *p* = 0.408, $\eta_{\text{p}}^{2}$ = 0.004. A significant two-way interaction between self-presentation instructions and attitudinal profile, *F*(3.067,683.967) = 54.835, *p* < 0.001, $\eta_{\text{p}}^{2}$ = 0.197, indicated different levels of expressed SA toward the different perceivers across self-presentation conditions (see Figure 1B). Simple effects analysis showed that, in the self-enhancement condition, those participants who were matched with the subjectively ambivalent perceiver reported a significantly higher level of SA relative to the baseline (*p* < 0.001), whereas those matched with the objectively ambivalent and the univalent positive perceivers reported a lower level of SA (*p*s < 0.001). This indicates that when people aimed to enhance their social image, they expressed SA only when it resembled the experienced attitudinal conflict of their perceiver. A reversed pattern emerged in the self-depreciation condition, in which those who were asked to generate a negative image on the subjectively ambivalent perceiver deviated from his/her experience of conflict by reporting a significantly lower level of SA than the baseline (*p* = 0.007). In the case of those participants who were matched with the univalent positive perceiver and the objectively ambivalent perceiver, they expressed significantly higher SA relative to their baseline (*ps* ≤ 0.018). Therefore, the expression of SA also aimed to project a negative social image on those who expressed a clear univalent stance or who held a (structurally) ambivalent attitude but did not experience it as conflicting. From this subset of findings, it is important to highlight the distinct self-presentation strategies that people used with the objectively and the subjectively ambivalent perceivers. They presumably indicated that people inferred different similarity–dissimilarity social preferences from these two ambivalent perceivers, based on the manipulated difference between them, this was the level of expressed attitudinal conflict (i.e., low vs. high SA).

##### Attitude valence

The self-presentation manipulation significantly affected the expressed attitude valence, *F*(1.587,707.932) = 314.778, *p* < 0.001, $\eta_{\text{p}}^{2}$ = 0.414, as opposed to the perceivers’ attitudinal profile, *F*(2,446) = 0.990, *p* = 0.372, $\eta_{\text{p}}^{2}$ = 0.004. However, the effect of self-presentation was moderated by the perceiver’s attitudinal profile (see Figure 1C), as indicated by the significant two-way interaction between both factors, *F*(3.175,707.932) = 28.936, *p* < 0.001, $\eta_{\text{p}}^{2}$ = 0.115. Simple effect analyses indicated that in the self-enhancement condition, participants matched with the univalent positive perceiver expressed more positive attitudes relative to their baseline (*p* < 0.001). Those matched with the subjectively ambivalent perceiver expressed less positive attitudes relative to the baseline (*p* < 0.001), whereas the attitude valence of those matched with the objectively ambivalent perceiver did not differ (*p* = 0.99). In the self-depreciation condition, people reported more negative attitudes toward the three different profiles than the baselines (*p*s < 0.001). However, those matched with the univalent positive perceiver expressed the most negative attitudes relative to the ambivalent profiles (*p*s < 0.001), with no significant differences between these two (*p* = 0.221). As argued above, we observed that a similarity–dissimilarity effect occurred with the univalent perceiver. However, this corresponded to the expression of similar (vs. dissimilar) univalent (positive *or* negative) attitudes when the objective was to make a good (vs. bad) impression.

#### Ambivalence and Liking

In addition to participants’ self-presentation, we examined whether participants’ (ambivalent) attitudes influenced the evaluation of the interpersonal liking of the manipulated perceivers. Thus, the tested model included the measures of participant’s OA, SA, and attitude valence and the perceiver’s attitudinal profile as between-subject factor. As we expected the effect of participants’ ambivalence on interpersonal liking to differ across the perceiver’s attitudinal profile, our main focus was the two-way interactions of the participants’ attitude measures (i.e., OA, SA, and valence) with the perceiver’s attitudinal profile. Furthermore, we tested whether the predictive value of attitude valence differed based on the level of experienced ambivalence. Therefore, we further entered the two-way interaction between SA and attitude valence and the three-way interaction with the perceiver’s attitudinal profile. The measures of participants’ attitudes were z-standardized in order to avoid multicollinearity issues with the interaction terms.

The GLM analysis showed significant main effects of participant’s OA, *F*(1,434) = 17.397, *p* < 0.001, $\eta_{\text{p}}^{2}$ = 0.039, and the perceiver’s attitudinal profile, *F*(2,434) = 3.106, *p* = 0.046, $\eta_{\text{p}}^{2}$ = 0.014. There was also a significant interaction between the perceiver’s attitudinal profile and participant’s SA, *F*(2,434) = 14.676, *p* < 0.001, $\eta_{\text{p}}^{2}$ = 0.063. Specifically, participants’ SA only predicted the liking ratings of the subjectively ambivalent perceiver, β = 0.29, *t*(434) = 3.351, *p* < 0.001, 95% CIs [0.12, 0.46], showing an ambivalence-similarity pattern. Although liking toward the univalent positive, β = −0.16, *t*(434) = −1.802, *p* = 0.072, 95% CIs [−0.33, 0.01], and the objectively ambivalent perceivers, β = −0.08, *t*(434) = −0.936, *p* = 0.350, 95% CIs [−0.25, 0.09], was not significantly affected by participants’ SA, the negative directionality of the observed trends indicated that participants considered to some degree the dissimilarity of these two perceivers in the level of experienced attitudinal conflict when assessing their interpersonal liking. Moreover, we observed a significant interaction between attitude valence and the perceiver’s attitudinal profile, *F*(2,434) = 5.957, *p* = 0.003, $\eta_{\text{p}}^{2}$ = 0.027, which showed that the valence of participants’ attitudes positively predicted the interpersonal liking of the univalent positive perceiver in line with the classical attitude-similarity effect, β = 0.48, *t*(434) = 3.501, *p* < 0.001, 95% CIs [0.21, 0.74]. This was not the case for the objectively ambivalent, β = −0.15, *t*(434) = −1.743, *p* = 0.082, 95% CIs [−0.02, 0.33], nor the subjectively ambivalent perceivers, β = 0.11, *t*(434) = 0.923, *p* = 0.356, 95% CIs [−0.12, 0.34]. Any other main or interaction effect was not significant.

### Discussion

Study 1 offers evidence for the strategic expression of ambivalence in a self-presentation setting, where the perceiver’s attitudinal profile determined the extent to which people express ambivalence. When people aimed to generate a positive image on another person, they mirrored the attitudinal profile of their perceiver in a structural (i.e., overall valence and level of OA) and an affective level (i.e., level of SA), even when their perceiver was ambivalent. In contrast, when people intended to generate a negative image, they reversed their perceiver’s profile by either reporting an attitude contrary in valence—in the case of the univalent perceiver—or opposing levels of OA and SA—in the case of the objectively and subjectively perceivers. We also found evidence of similarity patterns when observing that participants’ SA positively predicted interpersonal liking toward a subjectively ambivalent other, whereas a congruent attitude valence predicted it toward a univalent other.

From these findings, we believe that participants made use of ambivalence in an attempt to create attitude similarity, but, more importantly, emotional similarity between them and their perceiver. This is particularly salient in the distinction between the subjectively and the objectively ambivalent perceivers, where emotional similarity could function as the social validation of the former’s experience of attitudinal conflict. Our results supported this rationale in two respects. First, people expressed more ambivalent attitudes and presented themselves as similarly conflicted when aiming to positively impress a person who experienced an attitudinal conflict; second, ambivalent individuals liked the perceiver who expressed a similar experience of attitudinal conflict. This suggests that ambivalent attitude holders could be positively perceived as sources of emotional understanding and support when sharing the unpleasant experience of conflict.

## Study 2

Study 2 examined whether people’s intention when expressing ambivalence is indeed the social validation of the attitudinal conflict experienced by ambivalent others. While it has been suggested that ambivalence functions as a cue of social competence when addressing a controversial topic (Pillaud et al., 2013, 2018), the expression of ambivalence may also be intended as a validating framework for those ambivalent others who experience an attitudinal conflict. The aim of Study 2 was to address this issue directly and, at the same time, offer a conceptual replication of Study 1.

A first minor divergence from Study 1 was the use of a different attitude object, namely, abortion—a topic known to be associated with ambivalence (Craig et al., 2002; Schneider et al., 2015)—, with the aim of making our findings more generalizable to other attitude objects. The second and most important modification was that the self-presentation paradigm included an additional task in every condition. This task consisted of the composition of an essay that reflected the participants’ personal opinion about abortion. Similar to the other attitude measures, this essay would ostensibly be shared with the perceiver, and therefore its composition allowed us to infer underlying self-presentation intentions. In each self-presentation condition, people composed their essay by choosing among several pre-tested statements that reflected either positive or negative evaluations toward abortion or statements that validated ambivalent attitudes. These “ambivalence-validating” statements referred to claims that normalized two-sided stances and, more broadly, the experience of ambivalence toward abortion. One might argue that the task constrained (or aided) participants in terms of their attitude expression. Despite this, this task offered three important advantages. First, it allowed simple and systematic quantitative analysis of the content of the essay. Second, it offered participants a channel to express attitudes with specific content, in contrast to Study 1. Third, since the manipulated attitudinal profiles did not include any essay, the task avoided that participants anchored their selection of statements to their perceiver’s selection, as could have occurred with the responses to the attitude measures used in Study 1.

As for the experimental design of this study, it did not differ from Study 1, beyond the addition of the new dependent measures related to the composition of the essay. Thus, we expected to replicate the self-presentation pattern found in Study 1. Moreover, we hypothesized that the perceiver’s attitudinal profile would determine how people composed their essay toward abortion. Specifically, we formulated different pre-registered hypotheses^2^ regarding the overall (ambi)valence of the essay through the use of statements in favor or against and the use of “ambivalence-validating” statements. Taking always the participants’ baseline essay as a reference, we predicted that those matched with an ambivalent perceiver would compose a more two-sided essay (i.e., higher number of positive *and* negative statements) to create a positive impression, whereas the essay would be more one-sided (i.e., higher number of positive *or* negative statements) when instructed to make a negative impression. The essay of those assigned to the univalent perceiver (in this study, a univalent negative perceiver) would be more negative in the self-enhancement condition, whereas it would become more positive in the self-depreciation condition. Additionally, we predicted that when interacting with an ambivalent perceiver, people would make more use of ambivalence-validating statements when asked to generate a positive impression. We expected this difference to be greater among those matched with the subjectively ambivalent (vs. objectively ambivalent) perceiver given that people could anticipate the perceiver’s need for receiving some validation of their experienced attitudinal conflict. In contrast, regarding the univalent negative perceiver, we predicted a lesser use of this kind of statements in the self-enhancement condition. In the self-depreciation condition, we expected a decrease in the number of ambivalence-validating statements without significant differences among perceiver’s attitudinal profiles.

### Method

#### Ethics Statement

As a conceptual replication, this study followed the same recommendations provided for Study 1 by the Ethics Review Board of the Faculty of Social and Behavioral Sciences (FMG) of the University of Amsterdam. All subjects gave written informed consent in accordance with the Declaration of Helsinki.

#### Participants

Based on the power analysis performed for Study 1, we decided to recruit 300 participants in two equally spaced batches (*n* = 150), following a sequential approach (Lakens, 2014). We recruited a first batch of 163 participants from the United States through the online platform Prolific Academic, who were compensated with 1.28 USD. Fourteen participants who did not complete the study or failed one of the two attention checks included in the study (i.e., “In this question, we want you to click six” and within the essay composition “Do not select this option. This is an attention check for random clickers.”) were excluded from the analyses. This resulted in 149 participants (50.3% female, *M*_age_ = 33.15, *SD*_age_ = 11.23, ranging from 18 to 75). Given that the first batch of collected data did not allow to run exactly equally spaced analyses, we estimated an adjustment of the alpha level at 0.0248 using spending functions (Lakens, 2014). With this adjustment, most of our hypotheses were supported; hence, we decided to stop our data collection.

#### Procedure

The study followed a similar procedure as in Study 1, with only two differences. First, we changed our measure of attitude valence for a semantic bipolar scale built with pairs of opposite adjectives (e.g., moral–immoral). This scale was added to the stimuli used for manipulating the perceiver’s attitudinal profile, with the univalent negative perceiver reporting overall negative scores and the objectively and subjectively ambivalent perceivers’ scores close to the midpoint (see Supplementary Material). Second, after responding to the measures of OA, SA, and attitude valence in each within-subject self-presentation condition, participants were asked to compose the essay that reflected their viewpoint about abortion. To do so, participants were offered to choose from a list of 15 different statements at least once and as many as they considered for expressing their personal view. According to a pre-test with a different sample (*N* = 115; see Supplementary Material), five of these statements reflected an opinion in favor of abortion (e.g., “Abortion is essential in the case of rape, where women should definitely have the choice of terminating their pregnancy”), another five an opinion against abortion (e.g., “Since life begins at conception, abortion is akin to murder as it is the act of taking human life.”), and the last five “ambivalence-validating” statements normalized the experience of conflicting thoughts, indecision, and mixed reactions toward abortion (e.g., “Abortion is a controversial topic that many people feel torn about.”). The offered statements seemed to represent well the individual opinion of our participants. In the baseline measure, people selected on average 6.30 statements (*SD* = 2.55, ranging from 1 to 13 statements), and when asked to report how adequately the selected statements represented their views about abortion in a scale from −3 (*not adequately at all*) to 3 (*very adequately*), the average selection of statements was considered significantly adequate (*M* = 1.82, *SD* = 1.19) in comparison with the midpoint of the scale, *t*(148) = 18.750, *p* < 0.001, *d* = 1.536.

After completing the tasks under each the different self-presentation instructions, participants were debriefed and informed about their compensation.

#### Measures

##### Objective and subjective ambivalence

We used the same measures of OA and SA (α = 0.849) as in Study 1.

##### Attitude valence

Our measure of attitude valence toward abortion consisted of four 7-point semantic bipolar scales built with pairs of opposite adjectives (−3—*immoral, harmful, wrong, irresponsible*, 3—*moral, beneficial, right, responsible*). The overall scale had a high internal consistency (α = 0.965), and therefore an average score was calculated for each participant.^3^

##### Message ambivalence

In a similar way to the measure of OA, we used the sums of positive and negative statements people selected for their essays in Thompson et al.’s (1995) formula, in order to compute the extent to which people’s essay expressed a one-sided or a two-sided message: 1/2 (Positive + Negative) − |Positive − Negative|. Higher scores would represent an essay with a high number of positive *AND* negative statements (i.e., two-sided essay), whereas lower scores would represent an essay with a high number of positive *OR* negative statements (i.e., one-sided essay).

##### Ambivalence validation

We measured the social validation of the perceiver’s ambivalence through the number of “ambivalence-validating” statements selected for the composition of the essay.

##### Message valence

We used the difference between the number of positive statements and the number of negative statements as measure of message valence. Hence, values above zero will mean a predominantly positive essay regarding abortion, whereas values below zero will mean a predominantly negative message regarding abortion.

### Results

#### Manipulation Checks

Three independent GLM analyses showed that participants distinguished their perceiver congruently to the manipulated attitudinal profiles. The perceivers significantly differed in how positive/negative, *F*(2,146) = 102.953, *p* < 0.001, $\eta_{\text{p}}^{2}$ = 0.585, clear/confused, *F*(2,146) = 116.870, *p* < 0.001, $\eta_{\text{p}}^{2}$ = 0.616, and confident/unconfident, *F*(2,146) = 119.192, *p* < 0.001, $\eta_{\text{p}}^{2}$ = 0.620, participants perceived their opinion toward abortion. As Table 2 summarizes, the univalent negative perceiver significantly held the most negative opinion, whereas the objectively ambivalent had a slightly positive opinion, and the subjectively ambivalent moved around the midpoint. In addition, the subjectively ambivalent perceiver was again the one with the most confused and the least confident stance, followed by the objectively ambivalent perceiver and finally the univalent negative, whom participants considered to hold a fairly clear and confident opinion. Every pairwise comparison reached significance (*p*s ≤ 0.014) after applying Bonferroni corrections.

**TABLE 2:** Estimated mean levels of manipulations checks used to describe the different perceiver’s attitudinal profiles – Study 2.

**Perceiver attitudinal profile**	**Positivity**	**Clearness**	**Confidence**
Univalent negative	-2.43 [-2.75, -2.11]	2.61 [2.21, 3.01]	2.64 [2.25, 3.03]
Objectively ambivalent	0.65 [0.34, 0.97]	0.13 [-0.26, 0.52]	0.28 [-0.11, 0.66]
Subjectively ambivalent	0.01 [-0.30, 0.32]	-1.69 [-2.08, -1.31]	-1.61 [-1.99, -1.23]

*In brackets, lower and upper bounds of 95% CIs. Scales were from -3 to 3.*

#### Ambivalence and Self-Presentation

To be consistent with the analyses in Study 1 and a better accounting of the within-subject variance, we deviated from the pre-registered repeated measures GLMs and tested our hypotheses through three independent mixed models. The pre-registered analyses offered the exact same results and are accessible through the Supplementary Material. Our main focus was again the moderating effect of the self-presentation instructions as a function of the perceiver’s attitudinal profile on the expression of OA, SA, and attitude valence. In every model, we entered the fixed effects of the perceiver’s attitudinal profile, the self-presentation conditions, and the two-way interaction between them. As a random factor, we included the participant’s ID. In this case, the model with OA as dependent measure was the one having a singular fit; therefore, we conducted a repeated measures GLM analysis without the random factor.

##### Objective ambivalence

**FIGURE 2:**
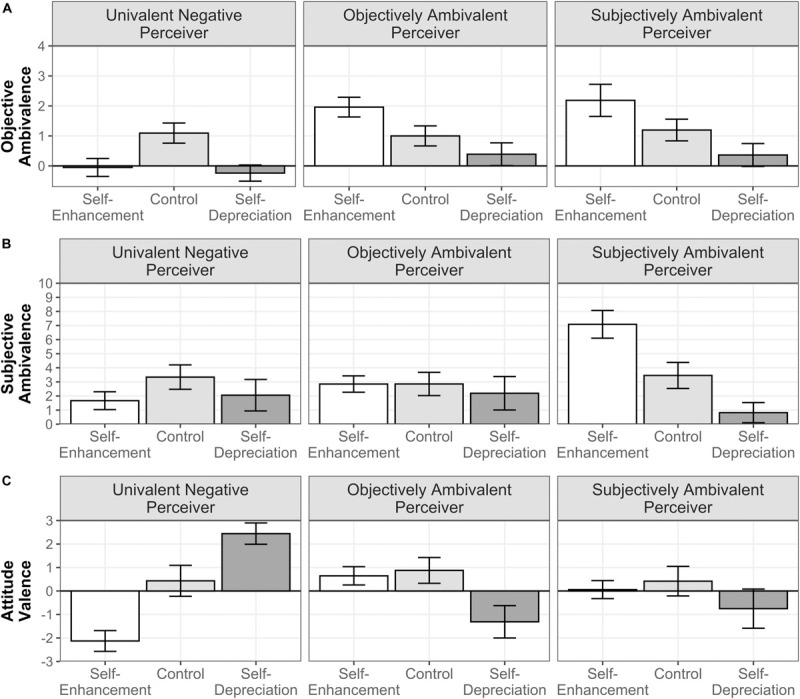
Moderated influence of self-presentation instructions by the perceiver’s attitudinal profile on reported OA **(A)**, SA **(B)**, and attitudinal valence **(C)** in Study 2. Error bars represent 97.52% CIs.

We found significant main effects of both self-presentation instructions, *F*(2,292) = 45.927, *p* < 0.001, $\eta_{\text{p}}^{2}$ = 0.239, and the perceiver’s attitudinal profile, *F*(2,146) = 34.070, *p* < 0.001, $\eta_{\text{p}}^{2}$ = 0.318. Furthermore, the two-way interaction between these two factors was significant, *F*(4,292) = 15.442, *p* < 0.001, $\eta_{\text{p}}^{2}$ = 0.175 (see Figure 2A). Simple effect analyses showed that in the self-enhancement condition, participants matched with the objectively and the subjectively ambivalent perceivers reported more OA relative to their baselines (*p*s < 0.001), but did not differ from each other (*p* = 0.99). Regarding the univalent negative perceiver, participants reported lower levels of OA (*p* < 0.001). In contrast, in the self-depreciation condition, OA decreased across profiles (*p*s ≤ 0.020), with the lowest OA reported toward the univalent negative perceiver, in comparison with the objectively and subjectively ambivalent perceivers (*p* = 0.012), and without significant differences between these two (*p* = 0.99). These results generally reproduce the similarity pattern observed in Study 1, with similarly ambivalent (univalent) attitudes to the ones expressed by the ambivalent (univalent) perceivers when the goal was to make a good impression and dissimilarly univalent attitudes when aiming at generating a bad impression. The only exception was the differences between the objectively and subjectively ambivalent perceivers in the self-enhancement condition, which plausibly did not emerge due to the smaller sample size of this study.

##### Subjective ambivalence

The two fixed effects of self-presentation, *F*(2,292) = 26.252, *p* < 0.001, $\eta_{\text{p}}^{2}$ = 0.110, the perceiver’s attitudinal profile, *F*(2,146) = 10.978, *p* < 0.001, $\eta_{\text{p}}^{2}$ = 0.049, and the two-way interaction, *F*(4,292) = 24.935, *p* < 0.001, $\eta_{\text{p}}^{2}$ = 0.191, were significant (see Figure 2B). Simple effects showed that, in the self-enhancement condition, participants who were matched with the subjectively ambivalent perceiver significantly increased their reported SA relative to their baseline (*p* < 0.001) and significantly decreased it in the self-depreciation condition (*p* < 0.001). Those matched with the univalent negative perceiver expressed significantly lower SA in the self-enhancement condition (*p* = 0.007), but a similar level in the self-depreciation condition relative to the baseline (*p* = 0.056). Participants expressing their SA toward the objectively ambivalent perceiver reported similar levels across self-presentation conditions (*p*s ≥ 0.65). These results indicate that the expression of SA was mainly modulated in the self-presentation toward the subjectively ambivalent perceiver, but not toward the univalent negative and the objectively ambivalent perceivers. While for the former, people assumed that a shared experience of SA would lead to a positive evaluation, for the latter expressing SA was not useful to enhance (or depreciate) their social image.

##### Attitude valence

The attitude valence significantly varied as a function of self-presentation conditions, *F*(2,292) = 14.121, *p* < 0.001, $\eta_{\text{p}}^{2}$ = 0.064, but not of attitudinal profiles, *F*(2,146) = 1.241, *p* = 0.29, $\eta_{\text{p}}^{2}$ = 0.006. We observed that these two factors significantly interacted, *F*(4,292) = 51.775, *p* < 0.001, $\eta_{\text{p}}^{2}$ = 0.333 (see Figure 2C). Simple effects indicated that for the univalent negative perceiver, participants reported significantly more negative attitudes in the self-enhancement condition (*p* < 0.001) and significantly more positive attitudes in the self-depreciation condition (*p* < 0.001) than their baseline. The attitudes expressed toward the objectively and subjectively ambivalent perceivers did not differ in the self-enhancement condition (*p*s ≥ 0.87), but they were significantly more negative in the self-depreciation condition (*ps* ≤ 0.002) relative to the baseline. As in Study 1, a similarity–dissimilarity pattern clearly emerged with the univalent perceiver, and to a certain degree with the ambivalent perceivers to which people expressed in the self-enhancement condition similarly in the middle ground (nor predominantly positive or negative), and more negative in the self-depreciation condition.

#### Essay Composition

Our second objective was to dig into participants’ intentions behind the similarity/dissimilarity self-presentation. For this purpose, we analyzed the selection of statements for the composition of essays that reflected their personal view about abortion. Table 3 summarizes the average number of positive, negative, and ambivalence-validating statements in each self-presentation condition and across the three perceiver’s attitudinal profiles. In order to test whether the interaction between these two factors influenced the message ambivalence, ambivalence validation, and overall attitude valence of the composed essay, we performed three mixed models including the same predictors as in previous analyses with these three new dependent measures.

**TABLE 3:** Average number of each type of statement selected in each self-presentation condition toward each perceiver’s attitudinal profile – Study 2.

	**Type of statement**			
**Experimental condition**	**Positive**	**Negative**	**Ambivalence-validating**	**Total**
**Control**				
Univalent negative	2.69 [2.06, 3.31]	1.25 [0.83, 1.67]	2.38 [1.86, 2.89]	6.31 [5.55, 7.08]
Objectively ambivalent	3.20 [2.62, 3.78]	1.10 [0.68, 1.52]	2.22 [1.76, 2.68]	6.52 [5.65, 7.39]
Subjectively ambivalent	2.61 [2.05, 3.16]	1.37 [0.89, 1.85]	2.08 [1.54, 2.61]	6.06 [5.19, 6.93]
**Self-enhancement**				
Univalent negative	0.40 [0.11, 0.68]	3.27 [2.73, 3.81]	0.75 [0.31, 1.19]	4.42 [3.82, 5.01]
Objectively ambivalent	2.32 [1.71, 2.93]	0.92 [0.47, 1.37]	2.86 [2.25, 3.47]	6.10 [5.17, 7.03]
Subjectively ambivalent	1.14 [0.66, 1.61]	0.88 [0.48, 1.28]	3.27 [2.67, 3.88]	5.29 [4.39, 6.20]
**Self-depreciation**				
Univalent negative	3.81 [3.32, 4.30]	0.38 [0.14, 0.61]	1.10 [0.60, 1.61]	5.29 [4.59, 6.00]
Objectively ambivalent	0.74 [0.34, 1.14]	2.88 [2.36, 3.40]	0.98 [0.50, 1.46]	4.60 [3.87, 5.33]
Subjectively ambivalent	1.22 [0.68, 1.75]	2.25 [1.66, 2.85]	0.33 [0.13, 0.53]	3.80 [3.19, 4.42]

*In brackets, lower and upper bounds of 97.52% CIs.*

##### Message ambivalence

**FIGURE 3:**
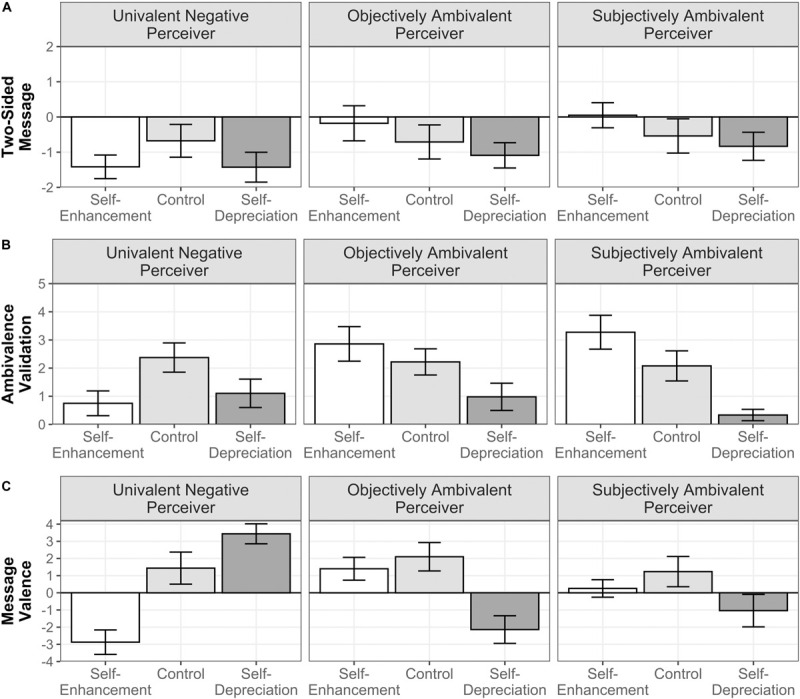
Moderated influence of self-presentation instructions by the perceiver’s attitudinal profile on message ambivalence **(A)**, ambivalence validation **(B)**, and message valence **(C)** in Study 2. Error bars represent 97.52% CIs.

The fixed effects of both self-presentation, *F*(2,292) = 13.033, *p* < 0.001, $\eta_{\text{p}}^{2}$ = 0.069, and the perceiver’s attitudinal profile, *F*(2,146) = 7.458, *p* < 0.001, $\eta_{\text{p}}^{2}$ = 0.041, as well as the two-way interaction turned out to be significant, *F*(4,292) = 6.268, *p* < 0.001, $\eta_{\text{p}}^{2}$ = 0.066 (see Figure 3A). Simple effect analyses showed that, relative to the baseline, those who were matched with the univalent negative perceiver composed a significantly more one-sided essay in the self-enhancement (*p* = 0.002) and the self-depreciation conditions (*p* = 0.002). For the objectively and subjectively ambivalent perceivers, participants showed significantly higher mean levels in the self-enhancement condition than in their baselines (*p* = 0.042 and *p* = 0.018, respectively), but no differences in the self-depreciation condition (*ps* ≥ 0.23). Note that a marginal increase in our OA measure, with mean values around zero, could imply that either the average essay was indeed more two-sided, or that the average essay did not make use of positive or negative statements. A descriptive look at the data suggested that for the two ambivalent perceivers, both explanations applied. In the self-enhancement condition, among those matched with the objectively ambivalent perceiver, there were 18% more essays that did not use positive or negative statements relative to the baseline, whereas 44% of the essays were more two-sided (or less one-sided). In the case of the group matched with the subjectively ambivalent perceiver, 37.25% more essays did not use positive and negative statements, whereas 33.33% of the essays were more two-sided (or less one-sided). Bear in mind that participants had to select at least one statement, meaning that those essays that did not include any positive or negative statements were instead including “ambivalence-validating” statements.

##### Ambivalence validation

The fixed effects of self-presentation, *F*(2,292) = 51.599, *p* < 0.001, $\eta_{\text{p}}^{2}$ = 0.206, and the perceiver’s attitudinal profile, *F*(2,146) = 5.407, *p* = 0.005, $\eta_{\text{p}}^{2}$ = 0.026, were significant, as it was the interaction between both factors, *F*(4,292) = 20.656, *p* < 0.001, $\eta_{\text{p}}^{2}$ = 0.172 (see Figure 3B). Simple effects indicated that, in the self-enhancement condition, participants matched with the univalent negative perceiver significantly decreased the number of ambivalence-validating statements relative to the baseline (*p* < 0.001). Importantly, this type of statements increased for the subjectively ambivalent perceiver (*p* < 0.001), and for the objectively ambivalent perceiver, even though in this last case the difference with the baseline did not reach significance (*p* = 0.077). Between these two ambivalent perceivers, the difference of ambivalence-validating statements was not significant (*p* = 0.51), although its directionality was congruent to what we predicted. In the self-depreciation condition, the number of ambivalence-validating statements significantly decreased across profiles (*p*s < 0.001) with only significantly less ambivalence-validating statements toward the subjectively ambivalent perceiver relative to the univalent negative perceiver (*p* = 0.035). The remaining comparisons between profiles were not significant. Overall, these results indicated that the validation of ambivalent stances toward abortion was as a self-presentation strategy toward the ambivalent perceivers, with certain signs suggesting that this validation was more evident in those essays directed to the subjectively ambivalent perceiver.

##### Message valence

The self-presentation manipulation significantly affected the overall valence of the essay, *F*(2,438) = 28.828, *p* < 0.001, $\eta_{\text{p}}^{2}$ = 0.116, whereas attitudinal profile did not exert any effect, *F*(2,438) = 1.792, *p* = 0.168, $\eta_{\text{p}}^{2}$ = 0.008. Their interaction turned out to be significant, *F*(4,438) = 59.408, *p* < 0.001, $\eta_{\text{p}}^{2}$ = 0.352 (see Figure 3C). Simple effects analysis showed that, whereas in the self-enhancement condition, the message valence did not significantly change for the objectively ambivalent (*p* = 0.42) and the subjectively ambivalent (*p* = 0.11) perceivers compared with their baselines, for the univalent negative perceiver, participants composed a more negative essay (*p* < 0.001). In the self-depreciation condition, the pattern reversed for the univalent negative perceiver, with the message of the essays being more positive than in the baseline (*p* < 0.001). Significantly more negative were the essays toward the objectively ambivalent (*p* < 0.001) and the subjectively ambivalent (*p* < 0.001), without these significantly differing between each other (*p* = 0.059).

### Discussion

The first set of results from Study 2 closely replicated the pattern observed in Study 1, with a different attitude object (i.e., abortion) and with a negative univalent profile. People mimicked the attitudinal profile of univalent and ambivalent perceivers when the goal was to make a positive impression on them, while adopting an opposing attitudinal profile when they aimed to make a negative impression. We also observed that people differentiated between ambivalent perceivers based on the latter’s level of experienced attitudinal conflict, using (avoiding) the expression of conflict to enhance (depreciate) their social image when addressing the subjectively ambivalent perceiver.

Study 2 further showed how people used the same self-presentation strategies when expressing their attitudes through the composition of an essay about abortion. For the univalent negative perceiver, we observed a clear switch in the participants’ essays from an overall negative message in the self-enhancement condition to an overall positive message in the self-depreciation condition. Regarding the ambivalent perceivers, participants generated a positive image by slightly balancing the number of statements expressing a positive and a negative stance, but mainly by focusing their overall message in statements that validated the holding of an ambivalence stance toward abortion, especially when the perceiver was subjectively ambivalent.

Although in general terms our predictions were supported, there were some hypothesized differences that our data did not confirm, such as the higher ambivalence validation in the case of the subjectively ambivalent perceiver relative to the objectively ambivalent perceiver. There are two methodological issues that could explain this. First, our sequential approach to data collection could have compromised our statistical power for detecting some expected between-subject differences. In addition, the application of conservative corrections for multiple comparisons saved us from potential type I errors, but could have also increased the probability of type II errors. Second, the measures chosen for analyzing the composition of the essay were perhaps not optimal. As we observed in the data, some differences between participants’ essays toward one or the other perceiver were not captured by the absolute number of one type of statements, but would have been by the relative use of one type versus the other (e.g., ambivalence-validating vs. positive and negative). Put differently, our analyses focused on the amount of information included in the essay and neglected potential differences in the emphasis of its message that the data descriptively suggest. Despite these limitations, we believe these results to be in line with the proposed rationale about why sharing ambivalence enhances interpersonal liking. When others express themselves as ambivalent, presenting oneself as similarly ambivalent while explicitly normalizing their ambivalent stance seemed to be an intuitive strategy that participants used for generating a positive social image. As previously discussed, this highlights not only the intention of socially validating the perceiver’s ambivalent attitude (in the case of the objectively ambivalent perceiver) but also their experience of attitudinal conflict (in the case of the subjectively ambivalent perceiver).

## General Discussion

Interpersonal liking relies to some extent on sharing similar attributes with others. In the attitudes domain, an extensive body of literature suggests that sharing a similar stance about a specific topic enhances interpersonal liking (e.g., Byrne et al., 1971; Singh et al., 2008). However, this so-called *attitude-similarity effect* has barely been studied in relation to ambivalent attitudes. The present research investigated the relationship between sharing ambivalent attitudes and interpersonal liking and offered evidence in support of a similarity effect previously neglected in attitude-similarity research. In our studies, people expressed similar (univalent) ambivalent attitudes to make a positive social image on (univalent) ambivalent perceivers (Studies 1 and 2). Moreover, people’s subjective ambivalence predicted the interpersonal liking toward a subjectively ambivalent other (Study 1). Importantly, the generated—or perceived—similarity observed in this research was not limited to the attitudinal structure (i.e., OA), but extended to the affective response of conflict associated with it (i.e., SA). Our research further suggests that sharing a similar experience of attitudinal conflict with others might indicate a disposition to socially validate the perceiver’s attitudinal conflict, as reflected in the attempts to normalize the holding of ambivalence when addressing ambivalent others (Study 2).

The unpleasant emotional state that ambivalent attitudes often elicit entails an important motivational factor that facilitates the interpretation of the obtained findings. People cope with their discomfort of feeling ambivalent by resolving the attitudinal inconsistency or by focusing on the reduction of that negative affective state (van Harreveld et al., 2009). Although the first option might be a more efficient strategy in the long term, people frequently find more immediate and effortless solutions for coping with the discomforting feeling of conflict (e.g., delay of the decision; Roster and Richins, 2009; van Harreveld et al., 2009). One of these solutions could be to interact with other ambivalent attitude holders that similarly experience the unpleasantness of being conflicted. Our research supports this notion by demonstrating that people express to be attitudinally conflicted to make a good impression on subjectively ambivalent others, and more directly, by showing that people’s SA predicts liking of subjectively ambivalent others.

These findings are aligned with previous research on emotional similarity. According to this line of work, if people realized that other individuals experience similar emotional reactions, they would not only find their emotional reaction to be socially validated (Anderson and Keltner, 2004), but they could also expect to receive more effective social support from someone who understands how they feel (Verhofstadt et al., 2008). Some studies have offered evidence of emotional similarity reducing individual stress in a shared threatening situation (i.e., public speech; Townsend et al., 2014). From these results, Townsend et al. (2014) argued that sharing emotional reactions with others might enhance certainty or predictability over the situation, which ultimately explains the decrease in levels of stress. Similarly, we argue that shared SA might be useful to decrease the discomforting experience of attitudinal conflict. Returning to the opening example of the current paper, if Robin learnt that Sam experienced his same feeling of conflict about nuclear disarmament, Robin would realize that his SA was not as unusual an emotional reaction as he would have thought. Since another person was experiencing the same kind of attitudinal incongruence (i.e., validating his SA), this could help to reduce his negative experience of conflict. From Sam’s perspective, if he knew that Robin felt conflicted toward nuclear disarmament, a potentially efficient strategy for enhancing his interpersonal liking would be to acknowledge that he also feels ambivalent, and that feeling ambivalent about nuclear disarmament is something common. In short, sharing ambivalence might be positively perceived when there is a motivation toward reducing the unpleasant experience of attitudinal conflict and, therefore, a good self-presentation strategy for enhancing interpersonal liking when interacting with an ambivalent other.

Although our interpretation of the link between ambivalence and interpersonal liking has mainly focused on its affective nature, this does not exclude the possibility that people strategically express ambivalence for other reasons. Ambivalent individuals might indeed be positively perceived based on their image of competence for offering pondered and balanced perspectives about controversial topics (Pillaud et al., 2013, 2018). Furthermore, a growing line of research highlights the benefits of ambivalence for cognitive and emotional regulation, such as increase of attention and creativity (Fong, 2006), less biased decision-making (Guarana and Hernandez, 2016), higher estimation accuracy (Rees et al., 2013), and more effective coping with outcome uncertainty (Plambeck and Weber, 2009; Reich and Wheeler, 2016). These attributes contribute to the overall social attractiveness of an ambivalent attitude holder depending on the context (e.g., deliberative vs. executive decision-making) and the fulfillment of the perceiver’s needs (e.g., resolution of attitudinal conflict).

This research is not exempt from some limitations. First, the presented evidence relies on survey data, collected in an online setting where participants did not expect to interact with their perceiver. Thus, our dependent measures did not capture actual behavior, but rather behavioral intention. Future research could replicate these results in an interactive, and more externally valid setting (e.g., real 1-to-1 interactions). Nevertheless, we believe that our setup offered experimental control over contextual modulators potentially present in real interactions (e.g., similarity–dissimilarity of other attributes, conversational dynamics, etc.), while still allowing to capture the strategic component of attitude expression. Furthermore, this research might be insufficient to draw firm conclusions about the underlying processes behind the social preferences of ambivalent attitude holders. In this regard, further research should investigate in more depth whether the social validation of subjective ambivalence has any effect on the endorsement of the ambivalent stance and, more importantly, the affective experience of attitudinal conflict. This would additionally shed light on the effectiveness of expressing ambivalence with the intention of enhancing one’s social image.

In addition, the extension of these results in applied contexts could lead to research questions with relevant practical implications. For example, it has already been proposed that shared ambivalence between leaders and followers in organizational settings relates to a better interpretation of the organizational context (Guarana and Hernandez, 2015). In their work, Guarana and Hernandez (2015) suggest that relational proximity between leader and follower increases the degree to which they share ambivalence about the context. Based on our findings, we wonder whether the identification of shared ambivalence could enhance relational (or interpersonal) proximity and, ultimately, not only improve the interpretation of the organizational context but also positively influence the work environment in the organization. In a similar vein, it would be interesting to examine whether sharing the experience of attitudinal conflict has positive effects on individual and interpersonal well-being. For example, in the domain of close relationships, previous research showed that (univalent) attitude similarity between partners is negatively associated with depression (Moore et al., 2017). It is plausible that the identification of those topics that partners feel similarly torn about could be good guidance for partner therapy to strengthen both the relationship and the partners’ well-being in it. Finally, our results might be relevant for the field of social communication. According to the present research, the understanding of the other’s state of attitudinal conflict is important when presenting oneself, but it might also become crucial when presenting others. Thus, related fields, such as political marketing and institutional and organizational communication—where often the goal is to enhance the social image of a political leader, an institution, or a company—, should consider that holding similarly ambivalent positions to their target audience might be an alternative strategy as a function of the context.

## Conclusion

Our research contributes to the ambivalence literature by suggesting that sharing ambivalent stances with others seems to have an important interpersonal function in the enhancement of one’s social image, and potentially in the psychological comfort of those who actually hold an ambivalent attitude that they experience as conflicting. Mechanisms of social validation, as proposed by work on attitude similarity, would explain why sharing ambivalence can also be a strategy that people follow for pursuing interpersonal liking and why ambivalent attitude holders prefer others who experience a similar attitudinal conflict.

## Data Availability Statement

The datasets generated for this study are available on request to the corresponding author.

## Ethics Statement

The studies involving human participants were reviewed and approved by the Ethics Review Board of the Faculty of Social and Behavioural Sciences (FMG) of the University of Amsterdam. The patients/participants provided their written informed consent to participate in this study.

## Author Contributions

DT-F and FvH developed the conceptual framework of the investigation. All authors contributed to the study design. DT-F conducted the data collection for all experiments. DT-F performed the data analyses and the interpretation of the results, in collaboration with FvH and IS. DT-F drafted the manuscript. FvH and IS provided critical revisions. All authors approved the final version of the manuscript for submission.

## Conflict of Interest

The authors declare that the research was conducted in the absence of any commercial or financial relationships that could be construed as a potential conflict of interest.

## Footnotes

1 We decided to include only one univalent positive (vs. negative) profile to simplify our experimental design, and consequently to increase the statistical power of our analyses. Study 2 included a univalent negative perceiver.

2 The pre-registration of Study 2 can be accessed at: https://osf.io/w26uf.

3 In order to be consistent with the measures and results from Study 1, we deviated from the valence-similarity measure specified in the pre-registration (i.e., the difference between the perceiver’s valence and the participant’s valence), without this deviation affecting our results.
