# Supplementary material for: Ambivalence and Interpersonal Liking: The Expression of Ambivalence as Social Validation of Attitudinal Conflict
Source: Front Psychol. 2020 Sep 29;11:525301. doi: 10.3389/fpsyg.2020.525301 (PMC7550634; doi:10.3389/fpsyg.2020.525301)
Supplement: Supplementary file 1 [file Data_Sheet_1.docx]

Supplementary Material

# Manipulation of Perceiver’s Attitude Profile – Study 1.

**Univalent Positive**

**
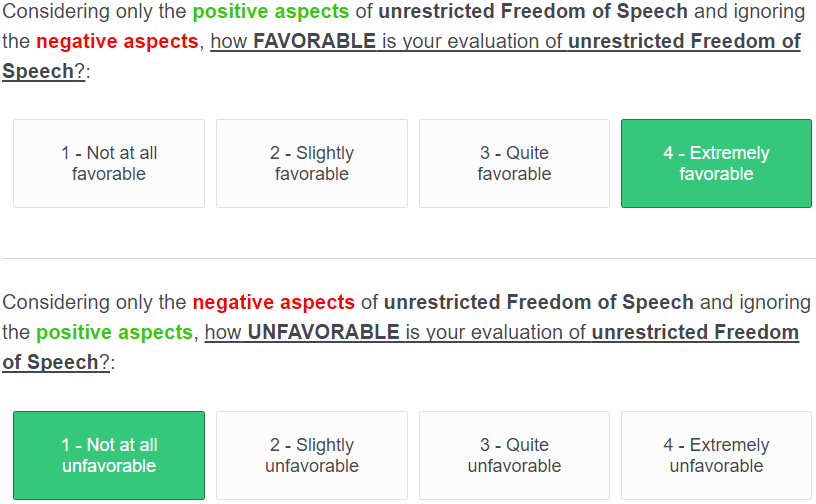
**

**
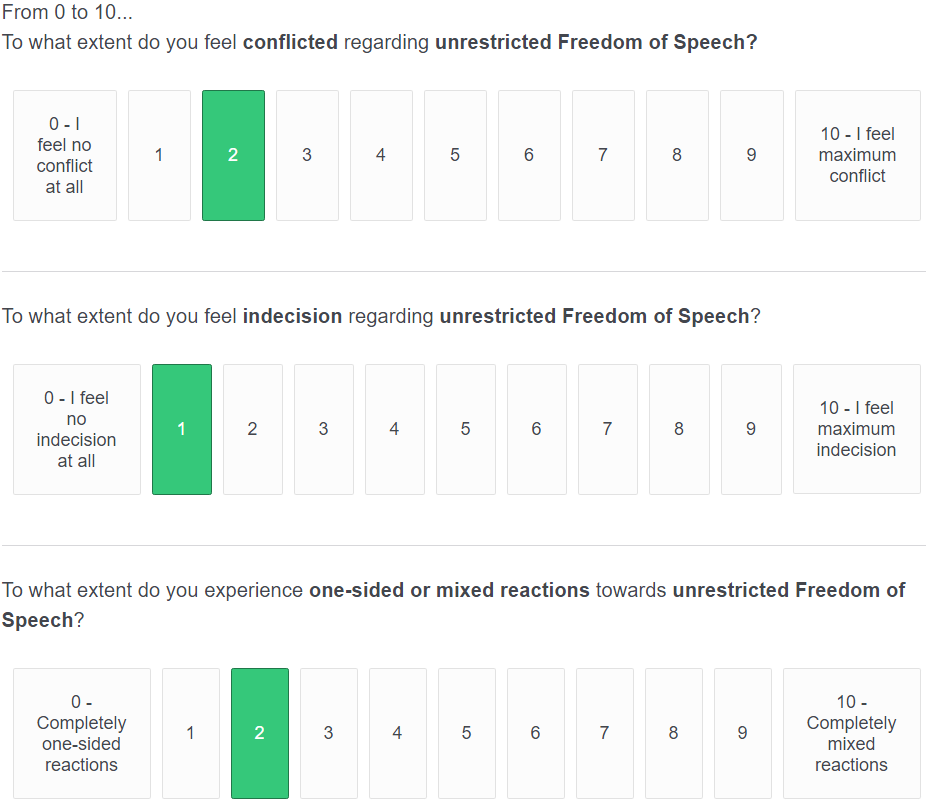
**

**Objectively Ambivalent**

**
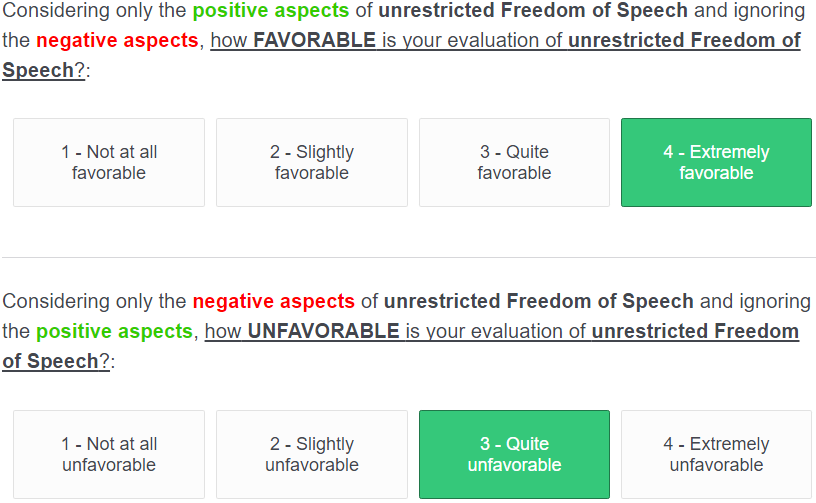
**

**
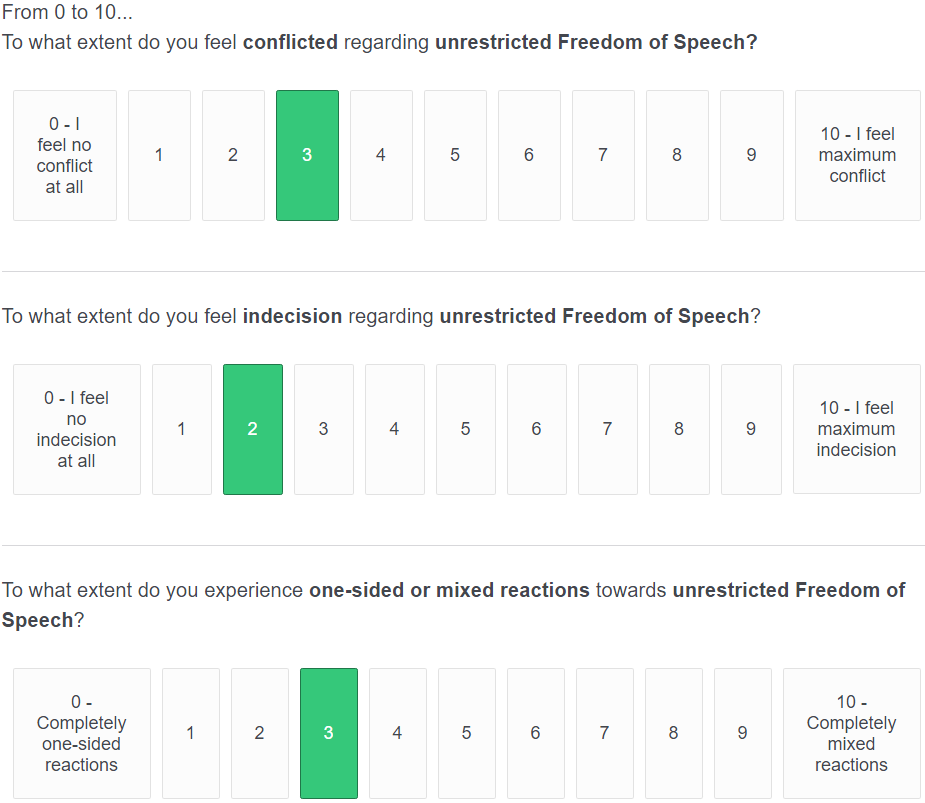
**

**Subjectively Ambivalent**

**
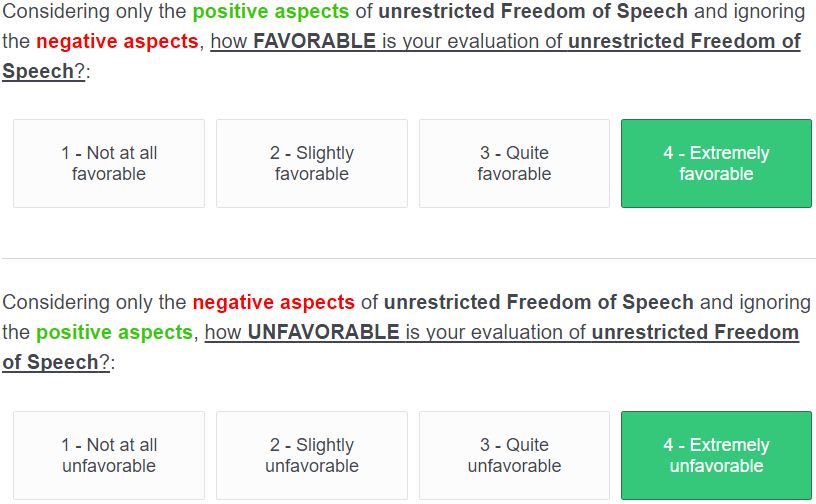
**

**
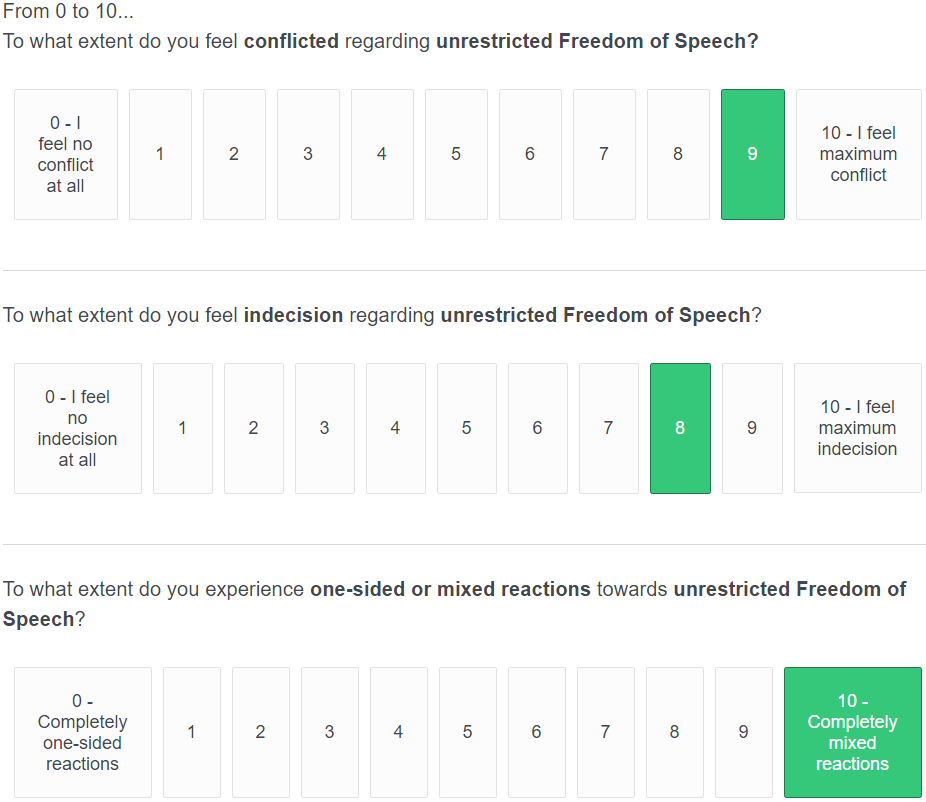
**

# Exploratory Variables in Study 1.

## Interest in Discussing

Our measure of interpersonal liking originally included the item “How much would you want to discuss unrestricted freedom of speech with this person?” (1 – *I do not want to discuss it at all,* 7 – *I really want to discuss it*). However, the internal consistency between this item and our dependent measure of liking was not higher than α = .522 across the different perceiver’s attitudinal profiles. Therefore, we independently considered this item as a measure of *interest in discussing* unrestricted freedom of speech.

## Relative Knowledge

Based on previous research (Sawicki et al., 2013; see also, Wallace et al., 2019), we considered of exploratory relevance to measure the participant’s relative knowledge about unrestricted freedom of speech. Two 7-point items measured participants’ relative knowledge about the attitudinal object in comparison to the perceiver (i.e., “How knowledgeable do you think this person is about unrestricted freedom of speech relative to you? To what extent do you think this person has knowledge about unrestricted Freedom of Speech that adds to yours?; 1 – *He/She is extremely more knowledgeable than me, His/her knowledge could really add to mine*; 7 – *I am extremely more knowledgeable than him/her*, *His/her knowledge could not add at all to mine*). The scale showed moderate levels of internal consistency (αs = .34-.72), so we considered them independently for any analysis.

## Perceiver’s Instrumentality

A 7-point item was included to measure the perceived instrumentality of the perceiver in providing useful insight about unrestricted Freedom of Speech (i.e., To what extent do you think this person could give you useful insight about unrestricted Freedom of Speech?; 1 – *I do not think at all that this person could give me useful insight,* 7 – *I really think that this person could give me useful insight*).

## Emotional Closeness

Another variable that we considered potentially informative regarding the perceiver’s interpersonal liking was the emotional closeness that participants could perceive on the different perceivers. We used the *Inclusion of Other in the Self Scale* (IOS; (Aron et al., 1992), a 7-point scale consisting of different illustrations of two circles that represented the participant and the target. Participants were asked to report the extent to which they thought they were emotionally close to the target. The shorter the distance between circles, the more emotionally close they perceived themselves relative to the target.

# Manipulation of Perceiver’s Attitude Profile – Study 2.

**Univalent Negative**


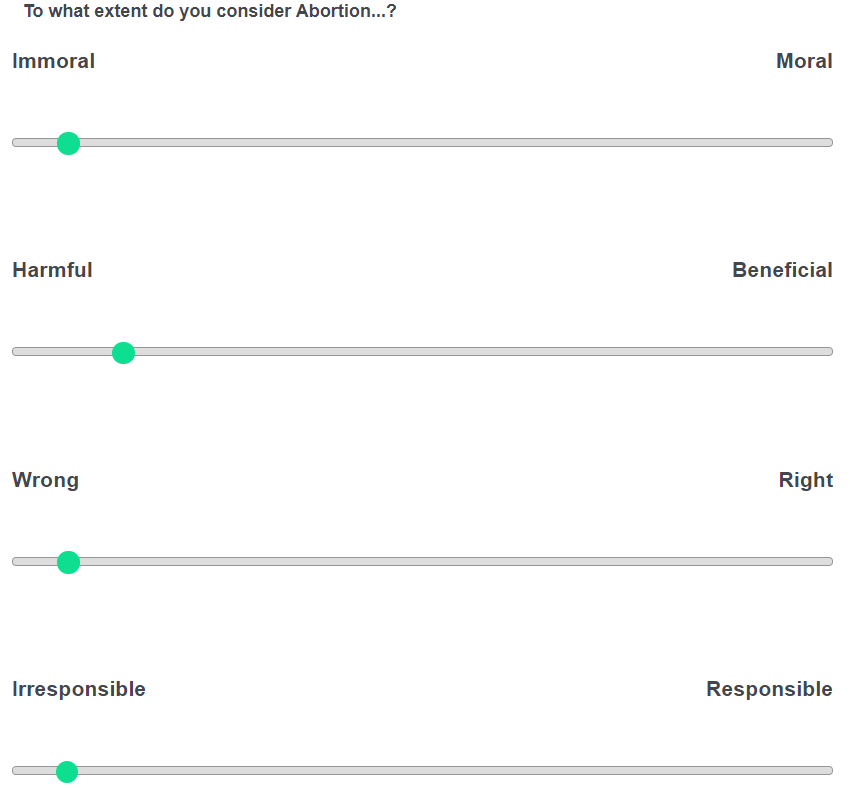


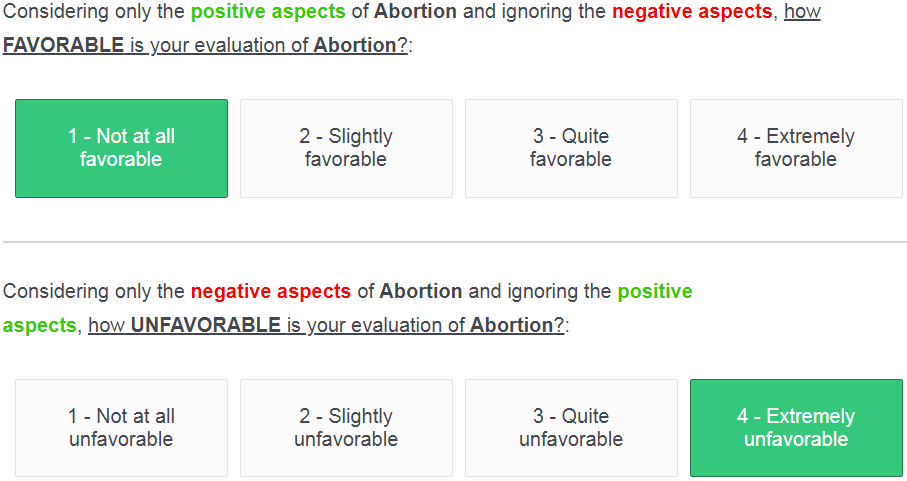


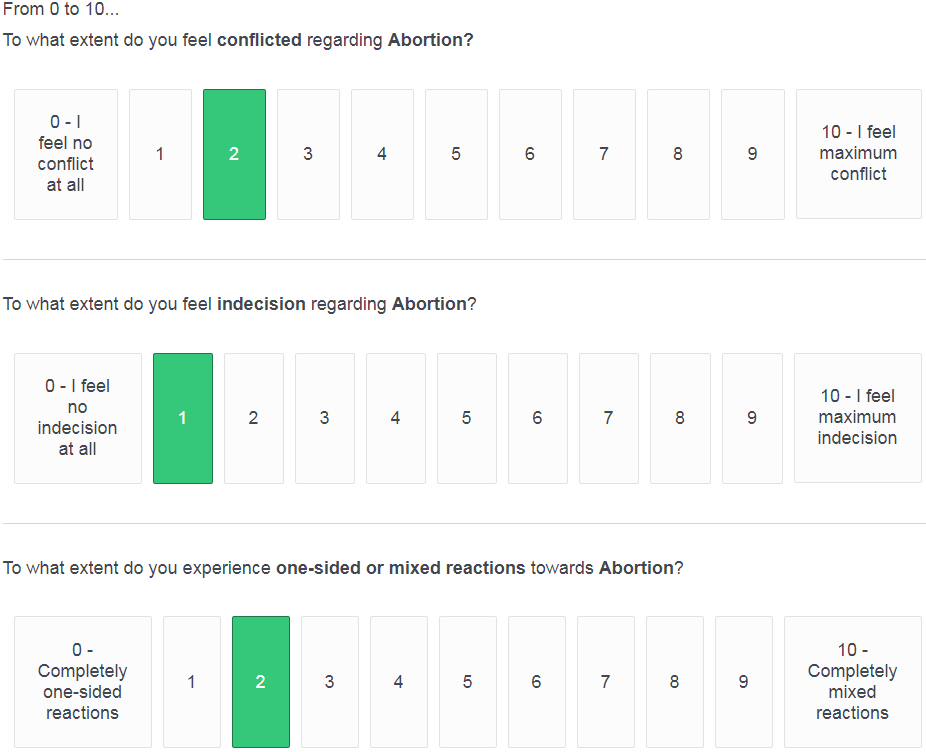


**Objectively Ambivalent**

**
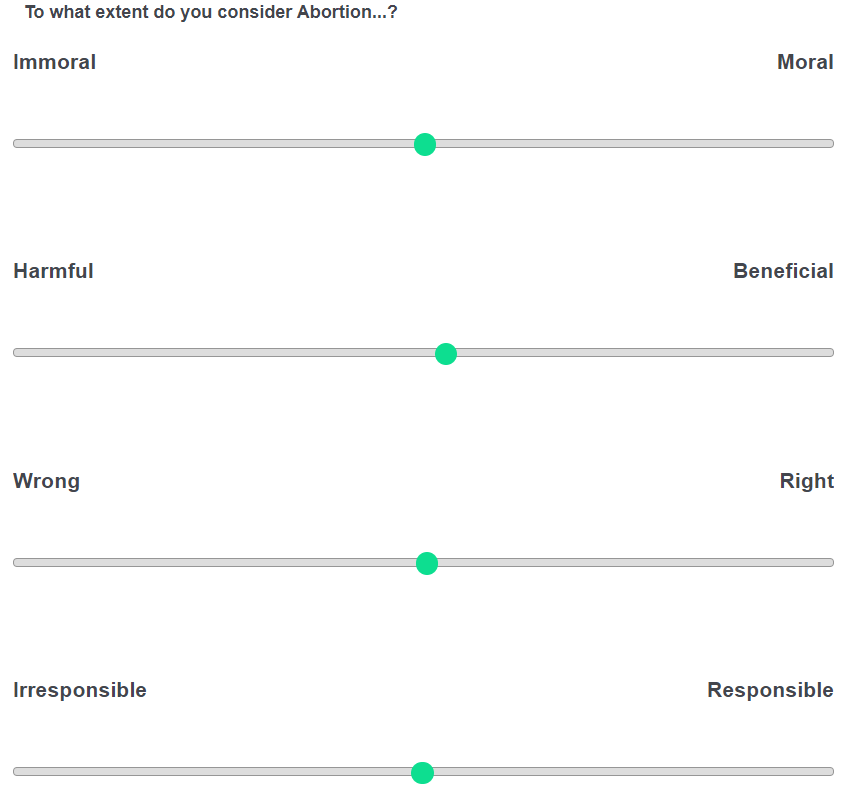
**

**
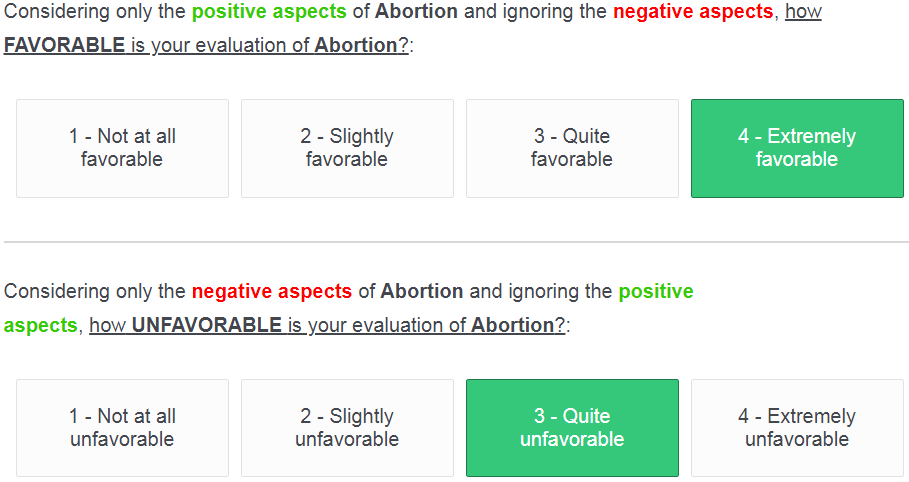
**

**
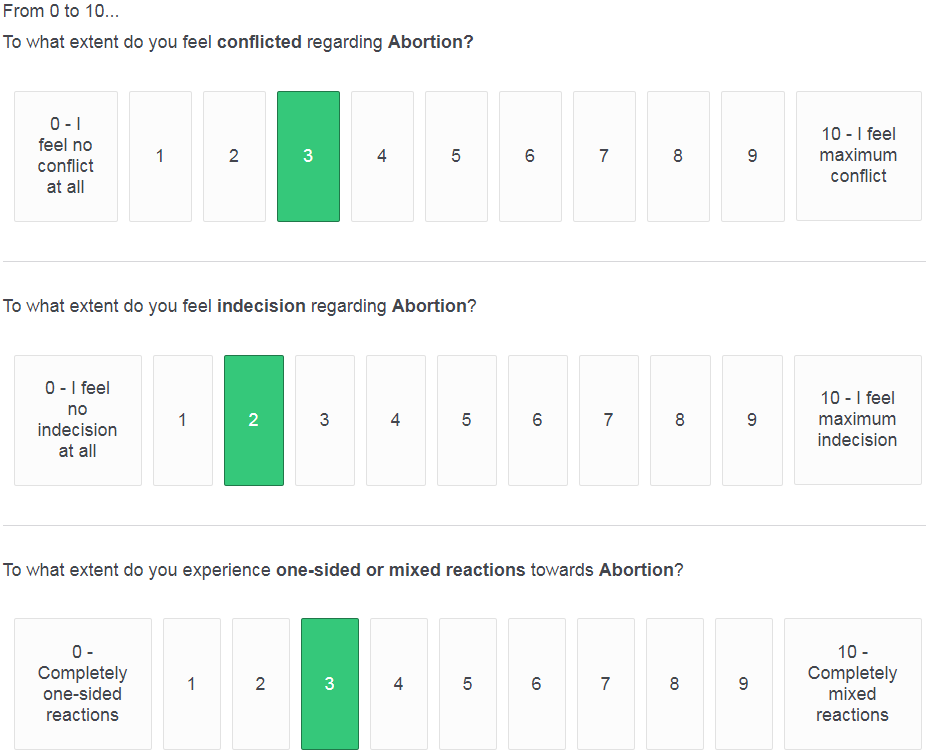
**

**Subjectively Ambivalent**

**
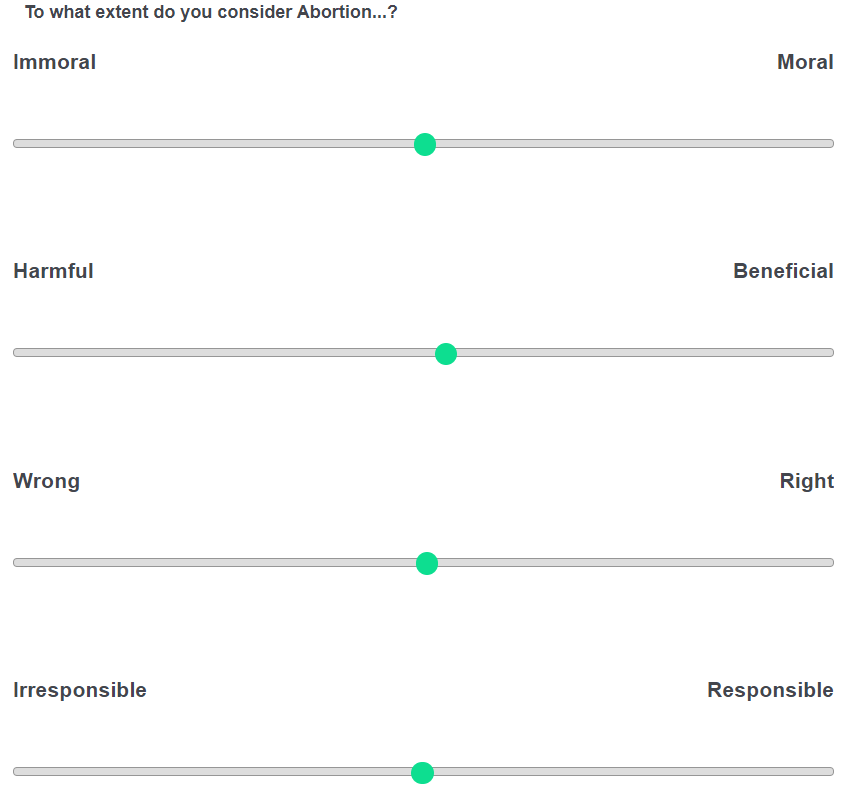
**

**
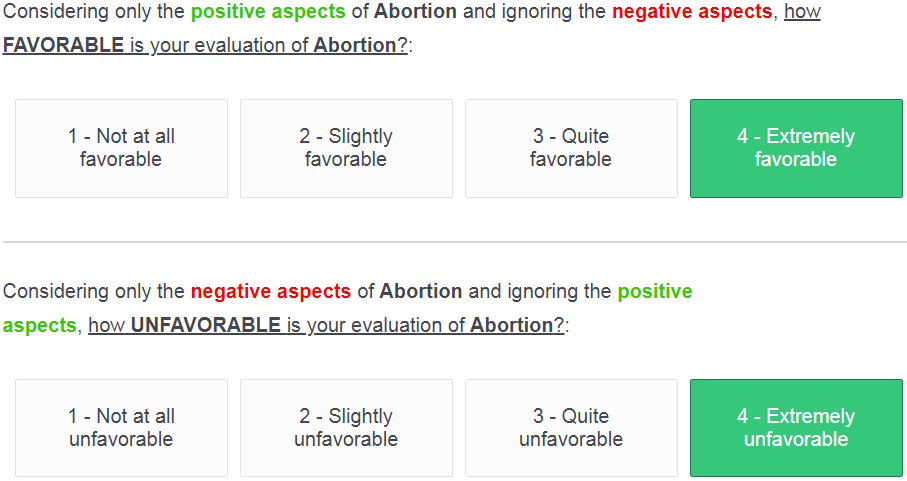
**

**
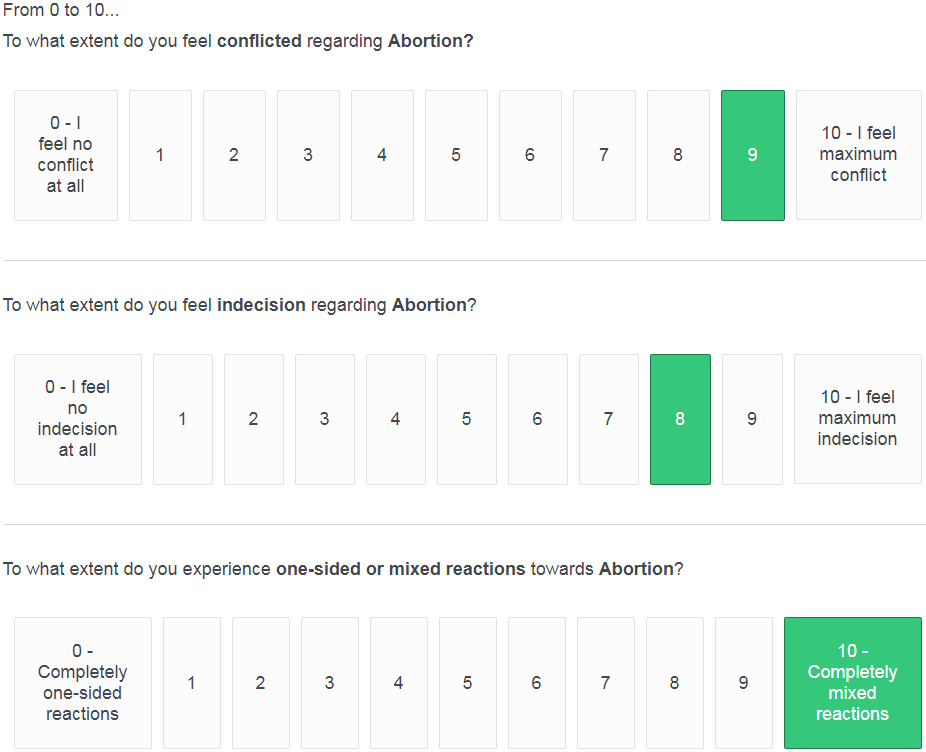
**

# Pre-test of Statements used for the Essay Composition – Study 2.

**Positive Statements**

**P1.** Nearly all abortions take place in the first trimester of pregnancy when a fetus cannot be regarded as a separate human entity as it cannot exist outside of the womb.

**P2.** Abortions have a low risk of serious complications and do not affect a woman's health or future ability to become pregnant or give birth.

**P3.** Abortion is essential in the case of rape, where women should definitely have the choice of terminating their pregnancy.

**P4.** The opportunity for a woman to have full control over her body is crucial to civil rights. Taking away women’s reproductive choice means to force them to continue undesired pregnancies, use contraception or undergo sterilization.

**P5.** Teenagers who do not have the choice to undergo abortion face grim prospects for the future. They are much more likely to leave school, receive inadequate prenatal care, and rely on public assistance to raise a child.

**Negative Statements**

**N1.** An abortion can result in medical complications, including not being able to become pregnant later in life.

**N2.** For women who demand complete control of their body, control should include preventing the risk of unwanted pregnancy through the responsible use of contraception or, if that is not possible, through abstinence.

**N3.** Those who choose abortions are often minors or young women with insufficient life experience to understand fully what they are doing.

**N4.** Most abortions are not reactions to cases of rape, but even in those cases human life should not be terminated.

**N5.** Since life begins at conception, abortion is akin to murder as it is the act of taking human life.

**Ambivalence-Validating Statements**

**A1.** Abortion is a controversial societal topic that many people feel torn about.

**A2.** Abortion is a complicated issue where people inevitably balance opposing interests and concerns.

**A3.** A substantial percentage of the public opinion is not able to identify as strictly pro-life or strictly pro-choice.

**A4.** Most people would agree that labelling abortion as right or wrong is a difficult judgment.

**A5.** Sensible people occupy middle ground on the issue of abortion.

**Pre-test Analyses:**

We recruited 115 US participants from Amazon Mechanical Turk who were asked to report the extent to which each statement reflected an opinion *in favor of abortion* and *against abortion*. Furthermore, they reported the extent to which the statement reflected that *it is common to have conflicted thoughts about abortion*, *it is common to feel undecided about abortion,* *it is common to experience mixed reactions towards abortion.*

Every item used a scale from -3 (Strongly Disagree) to 3 (Strongly Agree), being zero the midpoint of the scale. We conducted one-sample t-tests with each statement to confirm that the statements belonging to each category (i.e., positive, negative, ambivalence-validating) respectively expressed an opinion in favor or against abortion, or they normalized the holding of an ambivalent stance. Table 1 summarizes the results. Overall, people agreed that the positive statements expressed an opinion in favor of abortion, that the negative statements expressed an opinion against abortion, and that the ambivalence-validating statements expressed that it was common to have conflicting thoughts, feel undecided, and experience mixed reactions towards abortion.

| **Table 1. One-Sample t-tests comparing the mean of each statement with the midpoint of each scale.** | | | | | | | | | | | | | | | | | | | | |
| --- | --- | --- | --- | --- | --- | --- | --- | --- | --- | --- | --- | --- | --- | --- | --- | --- | --- | --- | --- | --- |
|  |  | **Opinion in Favor** | | |  | **Opinion Against** | | |  | **Conflicting Thoughts** | | |  | **Feeling Undecided** | | |  | **Mixed Emotions** | | |
| **Statement** |  | ***t*** | **df** | ***p*** |  | ***t*** | **df** | ***p*** |  | ***t*** | **df** | ***p*** |  | ***t*** | **df** | ***p*** |  | ***t*** | **df** | ***p*** |
| P1 |  | **10.370** | 110 | <.001 |  | -8.789 | 110 | <.001 |  | -3.945 | 110 | <.001 |  | -3.834 | 110 | <.001 |  | -3.114 | 110 | .002 |
| P2 |  | **12.410** | 111 | <.001 |  | -8.941 | 111 | <.001 |  | -5.140 | 111 | <.001 |  | -5.188 | 111 | <.001 |  | -4.568 | 111 | <.001 |
| P3 |  | **15.408** | 109 | <.001 |  | -10.179 | 109 | <.001 |  | -3.684 | 109 | <.001 |  | -3.881 | 109 | <.001 |  | -1.689 | 109 | .094 |
| P4 |  | **11.147** | 111 | <.001 |  | -10.561 | 111 | <.001 |  | -5.268 | 111 | <.001 |  | -7.141 | 111 | <.001 |  | -5.583 | 111 | <.001 |
| P5 |  | **10.276** | 110 | <.001 |  | -7.747 | 110 | <.001 |  | -3.655 | 110 | <.001 |  | -5.338 | 110 | <.001 |  | -4.061 | 110 | <.001 |
| N1 |  | -8.369 | 110 | <.001 |  | **12.463** | 110 | <.001 |  | -3.504 | 110 | .001 |  | -4.619 | 110 | <.001 |  | -3.844 | 110 | <.001 |
| N2 |  | -4.224 | 112 | <.001 |  | **5.045** | 112 | <.001 |  | -3.193 | 112 | .002 |  | -3.936 | 112 | <.001 |  | -3.181 | 112 | .002 |
| N3 |  | -6.763 | 110 | <.001 |  | **8.112** | 110 | <.001 |  | -2.746 | 110 | .007 |  | -3.984 | 110 | <.001 |  | -1.555 | 110 | .123 |
| N4 |  | -9.812 | 112 | <.001 |  | **13.144** | 112 | <.001 |  | -6.461 | 112 | <.001 |  | -7.993 | 112 | <.001 |  | -6.692 | 112 | <.001 |
| N5 |  | -10.845 | 110 | <.001 |  | **16.880** | 110 | <.001 |  | -7.789 | 110 | <.001 |  | -8.231 | 110 | <.001 |  | -7.820 | 110 | <.001 |
| A1 |  | -3.291 | 110 | .001 |  | -3.828 | 110 | <.001 |  | **12.852** | 110 | <.001 |  | **11.930** | 110 | <.001 |  | **14.260** | 110 | <.001 |
| A2 |  | -2.300 | 112 | .023 |  | -3.040 | 112 | .003 |  | **10.038** | 112 | <.001 |  | **11.470** | 112 | <.001 |  | **13.858** | 112 | <.001 |
| A3 |  | -3.776 | 109 | <.001 |  | -3.280 | 109 | .001 |  | **10.252** | 109 | <.001 |  | **8.623** | 109 | <.001 |  | **11.886** | 109 | <.001 |
| A4 |  | -2.521 | 110 | .013 |  | -4.521 | 110 | <.001 |  | **12.109** | 110 | <.001 |  | **13.038** | 110 | <.001 |  | **13.641** | 110 | <.001 |
| A5 |  | -1 | 111 | .402 |  | -2.091 | 111 | .039 |  | **8.204** | 111 | <.001 |  | **8.436** | 111 | <.001 |  | **11.716** | 111 | <.001 |

*Note.* In bold, the expected directionality for each group of statement.

# Pre-registered repeated measures GLM analyses for Attitude Measures and Essay Composition – Study 2.

| **Table 2. Repeated Measures GLMs with the Expression of Objective Ambivalence, Subjective Ambivalence and Attitude Valence as Criterion.** | | | | | | | | | | | | | | |
| --- | --- | --- | --- | --- | --- | --- | --- | --- | --- | --- | --- | --- | --- | --- |
|  | **Objective Ambivalence** | | | |  | **Subjective Ambivalence** | | | |  | **Attitude Valence** | | | |
| **Fixed Effects** | *df* | *F* | *p* | η_p_^2^ |  | *df* | *F* | *p* | η_p_^2^ |  | *df* | *F* | *p* | η_p_^2^ |
| **Between-Subject Effects** |  |  |  |  |  |  |  |  |  |  |  |  |  |  |
| Attitude Profile | 2 | 34.070 | <.001 | .318 |  | 2 | 10.978 | <.001 | .131 |  | 2 | 1.241 | .292 | .017 |
| Error | 146 |  |  |  |  | 146 |  |  |  |  | 146 |  |  |  |
| **Within-Subject Effects** |  |  |  |  |  |  |  |  |  |  |  |  |  |  |
| Self-Presentation | 2 | 45.927 | <.001 | .239 |  | 1.792 | 26.252 | <.001 | .152 |  | 1.639 | 14.121 | <.001 | .088 |
| Self-Presentation X Attitudinal Profile | 4 | 15.442 | <.001 | .175 |  | 3.584 | 24.935 | <.001 | .255 |  | 3.278 | 51.775 | < 001 | .415 |
| Error | 292 |  |  |  |  | 261.597 |  |  |  |  | 239.293 |  |  |  |
| *Note*. Degrees of freedom of the within-subject terms were corrected using Greenhouse-Geisser estimates when the sphericity assumption was violated. | | | | | | | | | | | | | | |

| **Table 3. Repeated Measures GLMs with the Composition of a Two-sided Essay, the Use of Ambivalence-Validating Statements, and the Valence of the Essay as Criterion.** | | | | | | | | | | | | | | |
| --- | --- | --- | --- | --- | --- | --- | --- | --- | --- | --- | --- | --- | --- | --- |
|  | **Two-sided Message** | | | |  | **Ambivalence Validation** | | | |  | **Message Valence** | | | |
| **Fixed Effects** | *df* | *F* | *p* | η_p_^2^ |  | *df* | *F* | *p* | η_p_^2^ |  | *df* | *F* | *p* | η_p_^2^ |
| **Between-Subject Effects** |  |  |  |  |  |  |  |  |  |  |  |  |  |  |
| Attitude Profile | 2 | 7.458 | .001 | .093 |  | 2 | 5.406 | .005 | .069 |  | 2 | 1.804 | .168 | .024 |
| Error | 146 |  |  |  |  | 146 |  |  |  |  | 146 |  |  |  |
| **Within-Subject Effects** |  |  |  |  |  |  |  |  |  |  |  |  |  |  |
| Self-Presentation | 2 | 13.033 | <.001 | .082 |  | 1.906 | 51.599 | <.001 | .261 |  | 1.910 | 28.734 | <.001 | .164 |
| Self-Presentation X Attitudinal Profile | 4 | 6.268 | <.001 | .079 |  | 3.811 | 20.656 | <.001 | .221 |  | 3.820 | 59.215 | <.001 | .448 |
| Error | 292 |  |  |  |  | 278.220 |  |  |  |  | 278.881 |  |  |  |
| *Note*. Degrees of freedom of the within-subject terms were corrected using Greenhouse-Geisser estimates when the sphericity assumption was violated. | | | | | | | | | | | | | | |
